# Harnessing Peptide Nucleic Acids and the Eukaryotic Resolvase MOC1 for Programmable, Precise Generation of Double-Strand DNA Breaks

**DOI:** 10.1021/acs.analchem.3c05133

**Published:** 2024-02-01

**Authors:** Gundra Sivakrishna Rao, Ahmed H. Saleh, Firdaws Melliti, Syed Muntjeeb, Magdy Mahfouz

**Affiliations:** † Laboratory for Genome Engineering and Synthetic Biology, Division of Biological Sciences, 4700 King Abdullah University of Science and Technology, Thuwal 23955-6900, Saudi Arabia

## Abstract

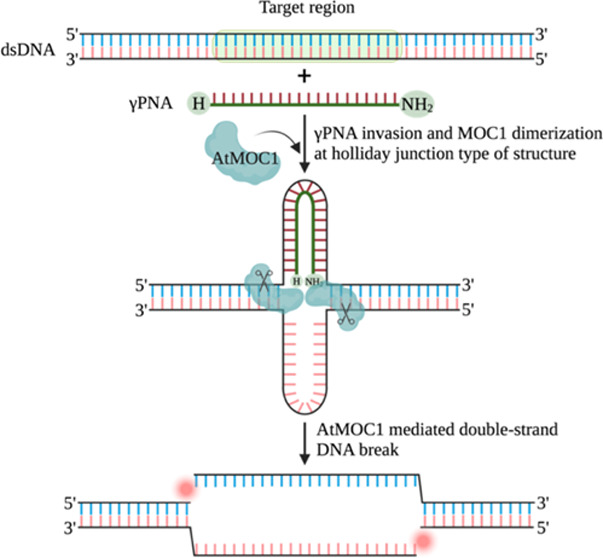

Programmable site-specific nucleases (SSNs) hold extraordinary promise to unlock myriad gene editing applications in medicine and agriculture. However, developing small and specific SSNs is needed to overcome the delivery and specificity translational challenges of current genome engineering technologies. Structure-guided nucleases have been harnessed to generate double-strand DNA breaks but with limited success and translational potential. Here, we harnessed the power of peptide nucleic acids (PNAs) for site-specific DNA invasion and the generation of localized DNA structures that are recognized and cleaved by the eukaryotic resolvase AtMOC1 from *Arabidopsis thaliana*. We named this technology PNA-assisted Resolvase-mediated (PNR) editing. We tested the PNR editing concept in vitro and demonstrated its precise target specificity, examined the nucleotide requirement around the PNA invasion for the AtMOC1-mediated cleavage, mapped the AtMOC1-mediated cleavage sites, tested the role of different types and lengths of PNA molecules invasion into dsDNA for the AtMOC1-mediated cleavage, optimized the in vitro PNA invasion and AtMOC1 cleavage conditions such as temperature, buffer conditions, and cleavage time points, and demonstrated the multiplex cleavage for precise fragment release. We discuss the best design parameters for efficient, site-specific in vitro cleavage using PNR editors.

Diverse natural molecular mechanisms have been harnessed to develop site-specific nucleases to enable precise in vivo gene editing, in vitro cloning of larger DNA fragments, and genome assembly applications. To date, zinc-finger nucleases (ZFNs), transcription activator-like effector nucleases (TALENs), and clustered regularly interspaced palindromic repeats (CRISPR)-associated proteins (Cas) systems have been developed and applied for myriad applications in basic and applied research.^1−4^ Among all, continuously evolving CRISPR-Cas technologies are highly predictable and reproducible and utilized widely for genome editing, cloning of larger DNA fragments, and pathogen detection.^5−12^ Despite the many recent developments in the CRISPR technology, the delivery of CRISPR reagents into organisms for genome editing remains a major bottleneck.^12^ The large protein size, highly negative charged phosphate backbone of sgRNA, and cell membrane barriers are the major delivery constraints.^13,14^ Moreover, the requirement of a 2–6 bp protospacer adjacent motif (PAM) also limits the application of CRISPR-Cas technologies for the in vivo PAM-independent editing and the in vitro PAM-independent generation of DSBs in a gene fragment for the downstream cloning applications. Therefore, the discovery of small site-specific nucleases would be a major priority to avoid the hurdles inherent in delivery into cells and PAM-independent target cleavage. CRISPR-independent technologies have been used to perform PAM-less genome editing.^15,16^ Argonaute-mediated genome engineering has shown promise for the generation of programmable DSBs. However, the use of these tools for genome editing in cells and organelles still needs to be evaluated.^16^

Resolvases are structure-selective endonucleases found in a wide variety of organisms, including bacteria, bacteriophages, archaea, and eukaryotes.^17,18^ Resolvases function in the recognition and resolution of four-way DNA intermediates called Holliday junction (HJ) structures.^19−23^ Resolvases dimerize and use dual active sites to catalyze two synchronized cuts at HJ complexes.^18^ Resolvases exhibit structure-dependent and structure-sequence-dependent cleavage specificities. Structure-dependent resolvases recognize and resolve the HJ structure.^24,25^ By contrast, sequence-specific and structure-dependent resolvases exclusively recognize the HJ structure and resolve it based on the nucleotide specificity around the HJ.^21−23,26,27^

The HJ resolvase monokaryotic chloroplast 1 (MOC1) was originally identified in *Chlamydomonas reinhardtii* and *Arabidopsis thaliana*, involved in homologous recombination and chloroplast nucleoid segregation during cell division.^23,28^ MOC1 proteins form dimers at HJ structures and symmetrically introduce nicks between the two cytosine (5′-C↓C-3′) residues.^23^ Yan et al.^29^ characterized different plant MOC1 proteins, including AtMOC1, GmMOC1, GrMOC1, NtMOC1, OsMOC1, and ZmMOC1 from *A. thaliana*, *Glycine max*, *Gossypium raimondii*, *Nicotiana tabacum*, *Oryza sativa*, and *Zea mays*, respectively. Each MOC1 exhibits a precise nucleotide specificity. Compared to the other MOC1 proteins, AtMOC1 displays symmetrical cleavage of 5′-C↓C-3′, 5′-C↓A-3′, and 5′-C↓G-3′ at the HJ structure after the cytosine molecule.^29^ Due to its varied nucleotide specificity, we selected AtMOC1 for the current study.

Peptide nucleic acids (PNAs) are synthetic analogues of nucleic acids.^30,31^ PNA molecules contain a neutral pseudopeptide backbone, which helps them bind to double-stranded DNA (dsDNA) and RNA with high affinity.^32^ The absence of phosphodiester and peptide bonds in PNA molecules makes them stable in different enzymatic environments. PNA molecules invade dsDNA in a sequence-dependent manner and form highly stable PNA–DNA duplex or triplex structures.^32,33^ γ-Modified tail clamp PNAs (γtcPNAs) have been used in vivo together with donor templates to correct genetic disorders.^34,35^ Biotinylated-γPNA molecules were employed as probes to invade specific dsDNAs and detect the target nucleic acids by CRISPR-Cas12b-based ssDNA cleavage.^36^ γPNAs have also been tested in vitro together with DNAzymes or Argonautes for the precise, programmable generation of double-strand DNA breaks (DSBs).^15,16^

The tiny sizes of resolvases and their unique ability for dsDNA nick formation at HJ structures in a sequence-dependent manner inspired us to exploit MOC1 proteins to generate site-specific DSBs for genome engineering applications. We hypothesized that PNA invasion would generate a localized DNA structure that mimics an HJ and serves as a substrate for resolvases, resulting in the generation of site-specific DNA DSBs. In this study, we relied on the functions of γPNA molecules to mediate invasion at specific dsDNA sites and generate the HJ mimicking structures and resolvase activity of AtMOC1 to specifically cleave these PNA-invaded structures. We named this technology PNA-assisted Resolvase-mediated (PNR) editing. We demonstrated that AtMOC1 specifically recognizes PNA-invaded DNA helix structures and generates programmable and precise dsDNA breaks. We also validated the nucleotide specificity of AtMOC1 around the γPNA invasion site, determined the cleavage sites of AtMOC1, evaluated the roles of different PNAs in AtMOC1 cleavage activity, examined γPNA target specificity via AtMOC1 cleavage, demonstrated precise fragment release by multiplex cleavage, and optimized the γPNA invasion and AtMOC1 cleavage reaction conditions in vitro.

## Experimental Section

### Construction of the AtMOC1 Expression Vector and Protein Purification

The pBASSY_AtMOC1 clone was a gift from Y.K. from Ibaraki University and N.Y. from Kyoto University. The *AtMOC1* gene fragment was excised from the pBASSY_AtMOC1 vector with the restriction enzymes NdeI and *Eco*RI and cloned into the pCold I plasmid (Takara Bio, Inc.) downstream of the 6xHis tag. The clones were confirmed by Sanger sequencing using pCold I primers (Table S1). The final protein expression vector was designated as pCold I_ AtMOC1 (Supporting file S1A). AtMOC1 protein purification was carried out as mentioned in the Supporting Methods.

### Design and Synthesis of γPNA and γtcPNA

The γPNA and γtcPNA molecules used in this study were tested in a previous study.^16^ All γPNA and γtcPNA molecules were custom synthesized by HLB Panagene Co., Republic of Korea. The γ-position in γPNA and γtcPNA was modified with the amino acid alanine (Table S3).

### Design and Cloning of PNA-Binding pUC19 and pMRS Targets

All γPNA and γtcPNA binding sequences were ordered from Integrated DNA Technologies, Inc. (IDT) as top and bottom oligonucleotides that produce *Bam*HI and *Eco*RI overhangs after annealing (Table S4). Each set of top and bottom oligonucleotides was phosphorylated, annealed, and ligated with the corresponding *Bam*HI- and *Eco*RI-digested pUC19 and pMRS vectors (Supporting Information, S2A,B). The ligated clones were confirmed by Sanger sequencing using the primers listed in Table S1.

### γPNA Invasion and Gel Mobility Shift Assay

γPNA invasion was carried out in 10 μL reaction volume including 1× MOPS buffer (20 mM MOPS pH 7.0, 5 mM CH_3_COONa, 1 mM EDTA). 2 μM γPNA molecules were treated with 20 nM of specific linear dsDNA target (609 bp) and incubated at 37 °C for 6 h. The dsDNA target was produced by PCR amplification of the pUC19 target containing PNA-binding regions, using 1447 forward and 1448 reverse primers listed in Table S1. The γPNA-invaded dsDNA reactions and the noninvaded dsDNA templates were separately mixed with 6× purple loading dye (NEB, B7024S) and run on a 6% native TBE gel at 120 V for 1 h 15 min. The gel was subsequently stained with 1× TBE buffer containing 1× SYBR Gold (Invitrogen, S11494) for <10 min and imagined in an iBright 1500 imaging system (Thermo Scientific, A44114).

### γPNA-Mediated Cleavage of Circular and Linear Plasmid Targets by AtMOC1

γPNA invasion and AtMOC1-mediated cleavage of circular or linear plasmid targets were achieved in two steps. In the first step, 200 ng of circular or XmnI-linearized pUC19/BsrGI-linearized pMRS targets were invaded by 250 nM of the desired PNA in a 10 μL reaction volume containing 1× MOPS buffer at 37 °C for 1 h (for the circular targets) and 6 h (for the linear targets). The invaded template was used for AtMOC1-mediated cleavage. The cleavage reaction was performed in a 20 μL reaction volume. The cleavage reaction mixture containing the PNA-invaded template (final concentration of 100 ng) or an equal concentration of noninvaded template, 1× NEB-rCutsmart buffer, 100 nM AtMOC1, and XmnI (only for the circular template) was incubated at 37 °C for 30 min. After adding 3 μL of 6× Purple Loading Dye (NEB, B7024S) to each sample, the samples were loaded onto a 0.9% (w/v) agarose gel containing GelRed and electrophoresed for 1 h and 30 min at 145 V. Finally, the gel was visualized in an iBright 1500 imaging system (Thermo Scientific, A44114).

All of the cleavage reactions were performed on ice. The reaction compositions and conditions were similar for all kinds of cleavage reactions performed in the current study. Minor changes were made based on the experiment. For example, the template was replaced with different templates for nucleotide composition experiments, different AtMOC1 protein concentrations were used for the protein concentration assay, PNAs were replaced with different types and lengths of PNAs, PNA concentrations were changed for the PNA concentration assay, different cleavage temperatures were tested for the temperature assay, and different time points were tested for PNA invasion and AtMOC1-mediated cleavage.

### Cleavage Site Identification

Circular pUC19 target-54 (containing γPNA binding sites) was independently invaded by γPNA1 and γPNA2. The γPNA-invaded templates were independently cleaved with AtMOC1 in the presence of XmnI, as described in the previous section for the cleavage reaction. The cleaved fragments were separated on a 0.9% agarose gel by gel electrophoresis. The released top and bottom fragments were separately eluted using a QIAquick gel extraction kit (Qiagen, Cat. no. 28706) and subjected to Sanger sequencing with 1448 reverse and 1447 forward primers, respectively (Table S1). The Sanger sequencing reads were analyzed using the SnapGene viewer.

### Cloning of the Multiplex Vector and AtMOC1-Mediated Multiplex Target Cleavage

For the multiplexing assay, we built pUC19 target-151, which can bind with two different γPNA molecules with a 1454 bp spacer sequence (Supporting file S2C). We initially ordered a γPNA1 binding target sequence as top and bottom oligos containing NdeI restriction sites at both ends from the IDT (Table S4). The annealed top and bottom oligos were cloned into the NdeI-linearized pUC19-49 vector containing a γPNA4 binding site: the resulting vector was named pUC19_γPNA1+γPNA4. A random 1.16kb fragment with *Eco*RI restriction ends was subcloned into *Eco*RI-digested pUC19_γPNA1+γPNA4 as a spacer. The resulting pUC19 multiplexing plasmid-151 was used for the AtMOC1-mediated multiplex cleavage experiments.

Circular and XmnI-linearized pUC19 multiplexing plasmid-151 were independently invaded by γPNA1 and γPNA4 in a 10 μL reaction as described above for plasmid invasion. AtMOC1-mediated multiplexing cleavage of circular and XmnI-linearized pUC19 plasmids was also performed, as described above for AtMOC1-mediated cleavage.

## Results and Discussion

### Overview of the PNR Gene Editing Concept

**Figure 1:**
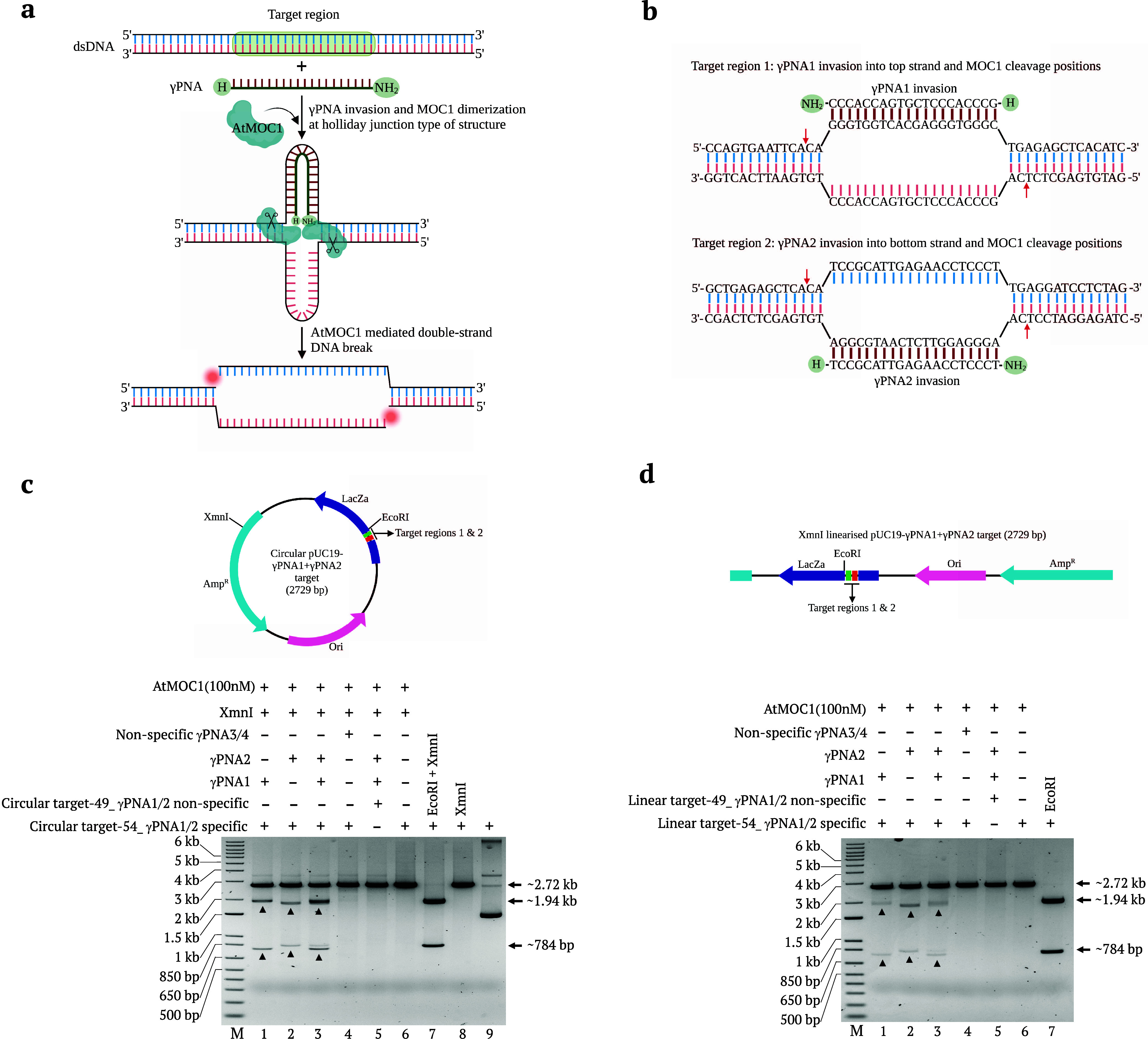
Proof of PNA-assisted AtMOC1-mediated cleavage of dsDNA. (a) Sketch providing an overview of PNA-guided AtMOC1-mediated double-strand break (DSB) formation. PNA invades the dsDNA in a sequence-specific manner and forms a loop. AtMOC1 recognizes and makes simultaneous nicks around the PNA invasion site, leading to the formation of DSBs. (b) Target regions 1 and 2 in pUC19 target-54 showing invasion of the top and bottom strands with γPNA1 and γPNA2 molecules, respectively. Arrows indicate the positions of AtMOC1 cleavage. (c) Gel image showing the AtMOC1-mediated cleavage of circular (γPNA-invaded, noninvaded, and nonspecific γPNA-invaded) dsDNA (Lanes 1–6). Restriction enzyme size controls included in Lanes 7 and 8. Lane 9 is the undigested plasmid control. XmnI was included in the circular plasmid cleavage reaction to release the fragment after AtMOC1-mediated DSB formation. A map of circular pUC19 target-54 with γPNA binding regions and restriction enzyme sites is included above the gel image. (d) Gel image showing the AtMOC1-mediated cleavage of XmnI-linearized (γPNA-invaded, noninvaded, and nonspecific γPNA-invaded) dsDNA (Lanes 1–6). Restriction enzyme size control included in Lane 7. A map of XmnI-linearized pUC19 target-54 with γPNA binding regions and restriction enzyme sites is included above the gel image. Lane M is the 1-kb plus DNA marker.

Resolvases have the ability to nick and relax complex HJ structures. PNA molecules exhibit sequence-specific invasion into dsDNAs. We presumed that PNA-invaded dsDNA structures might mimic the HJ structure that can be recognized by the resolvases and produce DSBs. In this study, we combined the invasion efficiencies of PNA molecules with the structure-dependent resolvase activity of AtMOC1 to obtain programmable DSBs in vitro (Figure 1a). We initially confirmed the ability of different γPNAs to invade the dsDNA targets. We amplified different dsDNA templates containing complementary regions of γPNA1–4 molecules. The amplified dsDNA templates were separately invaded by the γPNA1–4 molecules. A gel mobility shift assay showed that the γPNA1–4 molecules invaded the corresponding dsDNA templates with variable efficiencies (Figure S1a,b). Mobility shift assays also confirmed the sequence-specific invasion of γPNA molecules into dsDNA targets. We further explored the resolvase cleavage activity of eukaryotic AtMOC1 on γPNA-involved dsDNA targets. Figure 1a depicts the complete scheme of sequence-specific invasion of γPNA into dsDNA and the precise AtMOC1-mediated creation of DSBs.

### AtMOC1-Mediated Cleavage of Circular and Linear dsDNA Targets

We cloned the reannealed oligos containing γPNA1 and γPNA2 binding regions into the *Eco*RI and *Bam*HI restriction sites of pUC19. We cloned *AtMOC1* in the expression plasmid pCOLD I and expressed this gene in *E. coli* BL21DE3 cells. We purified the expressed AtMOC1 protein (25.1 kDa) as described in the Methods. To test the AtMOC1-mediated dsDNA cleavage concept, we used γPNA1, γPNA2, γPNA1+γPNA2, and nonspecific γPNA3+γPNA4 to separately invade the circular or XmnI-linearized plasmid targets (Figure 1b). We then employed the different γPNA-invaded templates for AtMOC1-mediated cleavage in NEB-rCutsmart buffer at 37 °C for 30 min. Our results show very precise cleavage of γPNA1, γPNA2, and γPNA1+γPNA2-invaded circular or linear targets, as they released the expected fragments (Figure 1c,d). By contrast, we did not detect any cleavage of noninvaded targets or in reactions containing a nonspecific target with specific γPNA1+γPNA2 or a specific target with nonspecific γPNA3+γPNA4, in circular or linear plasmids (Figure 1c,d). We demonstrated target-specific dsDNA invasion of PNA molecules and precise cleavage by AtMOC1. In PNR technology, target specificity is primarily obtained from γPNA molecules. Designing and synthesizing γPNA molecules is straightforward. Any region in the target genome can be targeted by PNR editors without a PAM requirement. Recently developed PANDA and PNP editors require the invasion of multiple PNA molecules into the target dsDNA and multiple nicking reagents to generate DSBs.^15,16^ By contrast, our PNR technology requires a single PNA molecule for dsDNA invasion and a nicking reagent to produce DSBs.

### Role of Nucleotide Composition around the γPNA Invasion Site in AtMOC1-Mediated Cleavage

**Figure 2:**
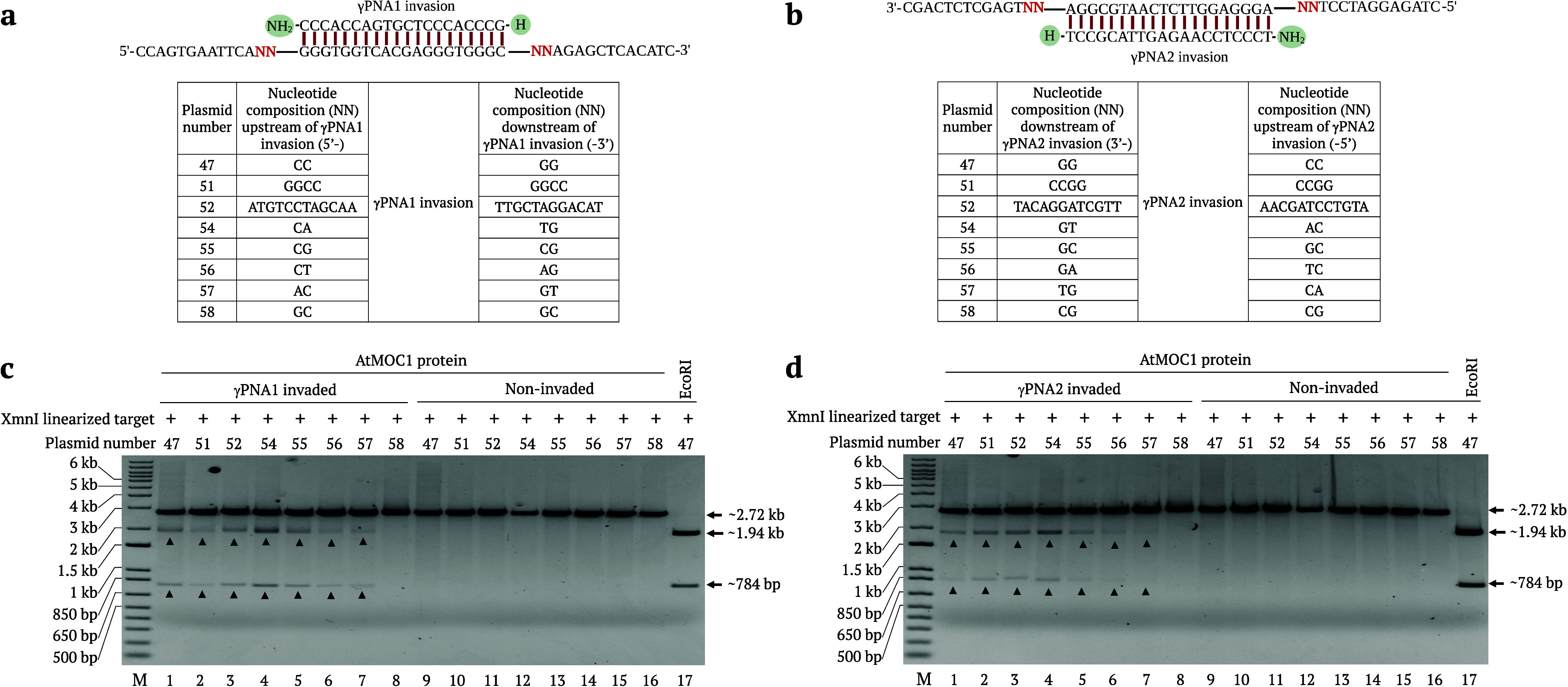
AtMOC1-mediated cleavage of different XmnI-linearized targets with different nucleotide compositions around the γPNA1 and γPNA2 invasion sites. (a, b) Tables showing the nucleotide compositions at both ends of the γPNA1 and γPNA2 invasion sites in different pUC19 target plasmids. In the diagram, NN represents the nucleotides that are replaced with different nucleotides in different targets; the remaining pUC19 plasmid sequence is the same in all targets. (c, d) Gel images showing the AtMOC1-mediated cleavage of γPNA1- and γPNA2-invaded XmnI-linearized dsDNA, respectively (Lanes 1–8). Gel images also showing the cleavage of noninvaded XmnI-linearized targets (Lanes 9–16). Restriction enzyme size controls included in Lanes 17 in both gels. Lane M shows the 1-kb plus DNA marker.

AtMOC1 proteins cleave nucleotides specifically after the cytosine (5′-C↓C-3′, 5′-C↓A-3′, and 5′-C↓G-3′) residue around HJs and resolve the HJ structures in a nucleotide-dependent manner.^23,29^ We wondered whether the nucleotide composition around the PNA invasion site would also affect the specificity of the AtMOC1-mediated cleavage. To test this notion, we designed different targets containing different nucleotide compositions around the γPNA1 and γPNA2 binding regions (Figure 2a,b). We independently cloned several templates with different nucleotide compositions into pUC19 (Figure 2a,b). The XmnI-linearized targets with different nucleotide compositions (Plasmids −47, −51, −52, and −54 to −58) were invaded by γPNA1 and γPNA2 independently. We subjected the invaded and noninvaded targets to AtMOC1-mediated cleavage at 37 °C for 30 min. We observed differential AtMOC1-mediated cleavage in plasmids −47, −51, −52, and −54 to −57 around the γPNA invasion site and no cleavage of noninvaded plasmids −47, −51, −52, and −54 to −57 (Figure 2c,d). Interestingly, pUC19 target-58 did not show any cleavage. To confirm that the nucleotides around the γPNA invasion site in pUC19 target-58 indeed inhibit AtMOC1-mediated cleavage, we designed a few more targets containing the target-58 nucleotides at one end and different nucleotides at the other end of the γPNA invasion site (Figure S2a,b), followed by the analysis of AtMOC1-mediated cleavage. The presence of pUC19 target-58 nucleotides at one end of the γPNA invasion site did not affect the cleavage activity of AtMOC1 (Figure S2c,d). We employed additional targets with different nucleotide compositions one nucleotide away from the γPNA invasion site (Figure S3a). The newly designed targets also showed precise cleavage at the γPNA invasion site, irrespective of their nucleotide composition (Figure S3b). Our results demonstrated that AtMOC1 can recognize and cleave the PNA-invaded structure independent of the nucleotide composition. It will be essential to obtain a deeper structural understanding of AtMOC1 binding and the roles of nucleotides in the AtMOC1 cleavage of the γPNA-invaded targets.

### Identification of Cleavage Sites

**Figure 3:**
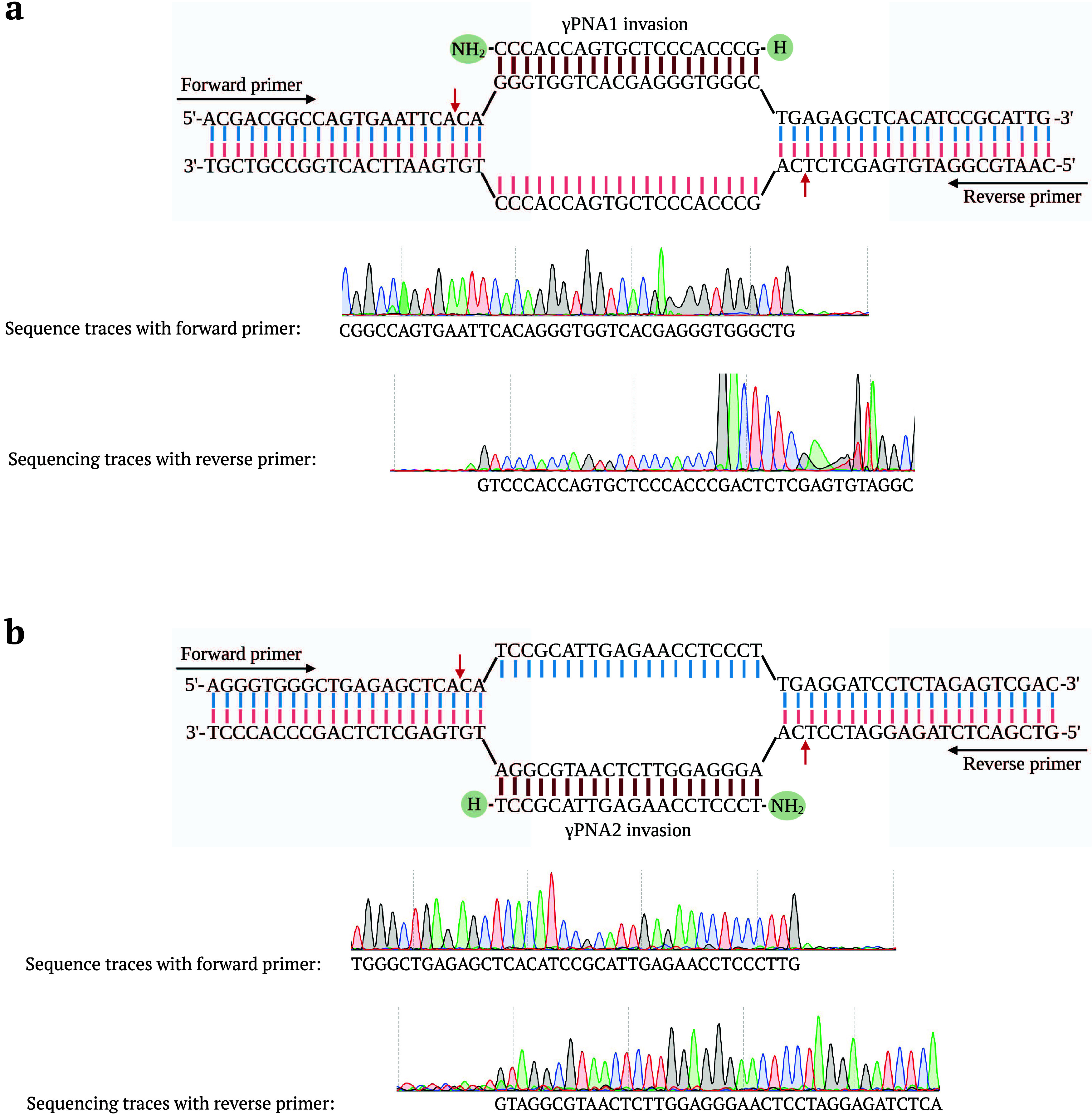
AtMOC1-mediated cleavage and cleavage site identification. (a, b) Sketches representing the separate invasions of γPNA1 and γPNA2 into the pUC19 target-54, respectively. The circular pUC19 target-54 was invaded by γPNA1 or γPNA2 and independently cleaved with AtMOC1 + XmnI. Both released fragments were sequenced separately with forward and reverse primers. The sequencing traces in the figures reveal precise AtMOC1-mediated cleavage at the 5′ ends and 2-nt away from the γPNA1 or γPNA2 invasion sites in the top or bottom strands.

Mapping cleavage positions is important for the downstream use of the cleavage products. To identify the cleavage positions of AtMOC1 at the γPNA invasion site, we invaded the circular pUC19 target-54 plasmid with γPNA1 (which invades the bottom strand) and γPNA2 (which invades the top strand), cleaved the invaded templates with AtMOC1 and the restriction enzyme XmnI, and separated the cleavage products by 1% agarose gel electrophoresis. We then subjected the top and bottom cleaved fragments from the two different γPNA invasion reactions to Sanger sequencing. Cleavage site mapping results revealed that AtMOC1 nicks the top strand 2-nt before the γPNA invasion site and the bottom strand 2-nt after the γPNA invasion site and collectively creates a DSB around the γPNA invasion site. We observed similar AtMOC1 cleavage patterns irrespective of the strand invaded (top or bottom) by the γPNA molecules (Figure 3a,b). We also detected an identical cleavage pattern for any template invaded (top or bottom strands) by γPNA. This cleavage pattern is consistent with the previously reported MOC1-mediated cleavage pattern of HJ structures, where the cleavage occurs at the 5′ end in the top strand before the HJ and 5′ end in the bottom strand after the HJ structure.^23,29^ Precise cleavage is extremely important for generating the correct DNA overhangs. Cleavage site mapping also demonstrated that PNR editors produce only 3′ overhangs upon cleavage. These results facilitate the design of PNR reagents for the downstream in vitro cloning of larger genomic fragments.

### Effects of Different Types of PNAs and Different Lengths of PNAs on the Cleavage Activity of AtMOC1

Validating different PNAs is essential for designing PNR editors for in vitro or in vivo site-specific cleavage. Here, we assessed the invasion abilities of different types of γPNA molecules. We initially tested γPNA3 and γPNA4 molecules by designing and generating pUC19 target-49, containing the γPNA3 and γPNA4 binding sequences (Figure S4a). The XmnI-linearized pUC19 target-49 was invaded by γPNA3, γPNA4, γPNA3+γPNA4, and nonspecific γPNA1+γPNA2 molecules (Figure S4b). We subjected the invaded, noninvaded, and nonspecific invaded targets to AtMOC1-mediated cleavage. AtMOC1 cleaved the γPNA-invaded target precisely, whereas no cleavage was observed in the noninvaded and not specifically invaded targets (Figure S4c). AtMOC1 displayed similar cleavage activities using all types of γPNA molecules.

**Figure 4:**
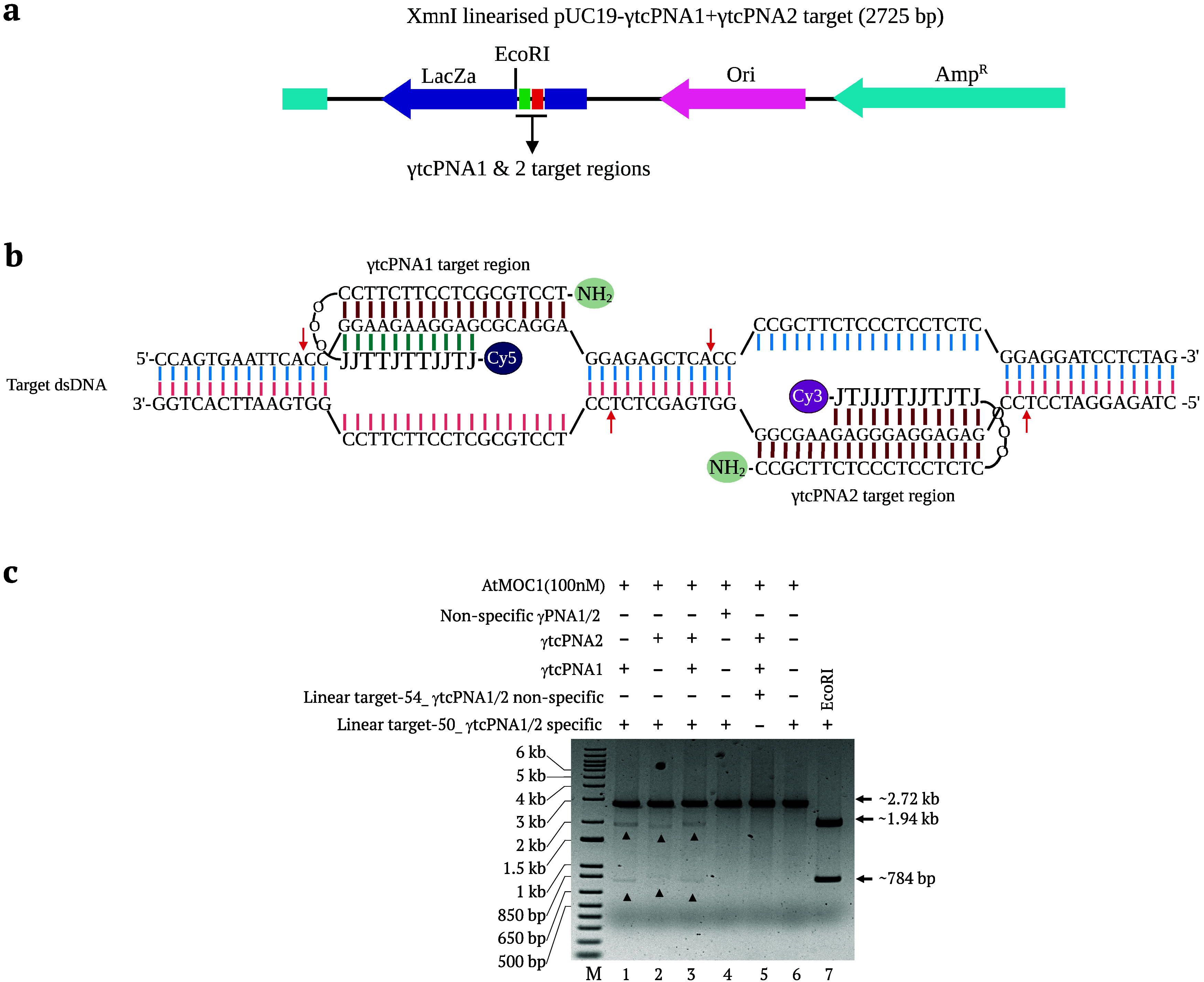
AtMOC1-mediated cleavage of γtcPNA-invaded XmnI-linearized dsDNA. (a) Map of XmnI-linearized pUC19 target-50 showing γtcPNA binding regions and restriction enzyme sites. (b) Diagram showing γtcPNA binding regions 1 and 2 in the pUC19 target-50 plasmid. Arrows indicate the positions of AtMOC1 cleavage. (c) Gel image showing the AtMOC1-mediated cleavage of XmnI-linearized (γtcPNA-invaded, noninvaded, and nonspecific γPNA-invaded) dsDNA (Lanes 1–6). Restriction enzyme size control included in Lane 7. Lane M shows the 1-kb plus DNA marker.

γ tail clamp PNAs (γtcPNAs) invade dsDNA and form triple-helix structures via Watson–Crick and Hoogsteen base pairing with the template DNA.^37^ We therefore investigated the AtMOC1-mediated cleavage of γtcPNA-invaded targets. We designed and cloned pUC19 target-50 containing γtcPNA1 and γtcPNA2 binding sequences (Figure 4a). The XmnI-linearized pUC19 target-50 was invaded by γtcPNA1, γtcPNA2, γtcPNA1+γtcPNA2 and nonspecifically invaded by γPNA1+γPNA2 molecules (Figure 4b). We treated different combinations of invaded, noninvaded, and nonspecifically invaded targets with AtMOC1. AtMOC1 specifically cleaved the γtcPNA1-, γtcPNA2-, γtcPNA1+γtcPNA2-invaded targets, whereas pUC19 target-50 invaded by nonspecific γPNA1+γPNA2, nonspecific pUC19 target-54 invaded by γtcPNA1+γtcPNA2, and noninvaded target did not show any cleavage (Figure 4c). We observed a sequence-specific invasion of γtcPNA and precise cleavage with AtMOC1. Overall, we demonstrated that the target specificity and type of PNA are not limiting factors for AtMOC1-mediated cleavage. AtMOC1 is compatible with different types of PNAs and displays precise target cleavage.

**Figure 5:**
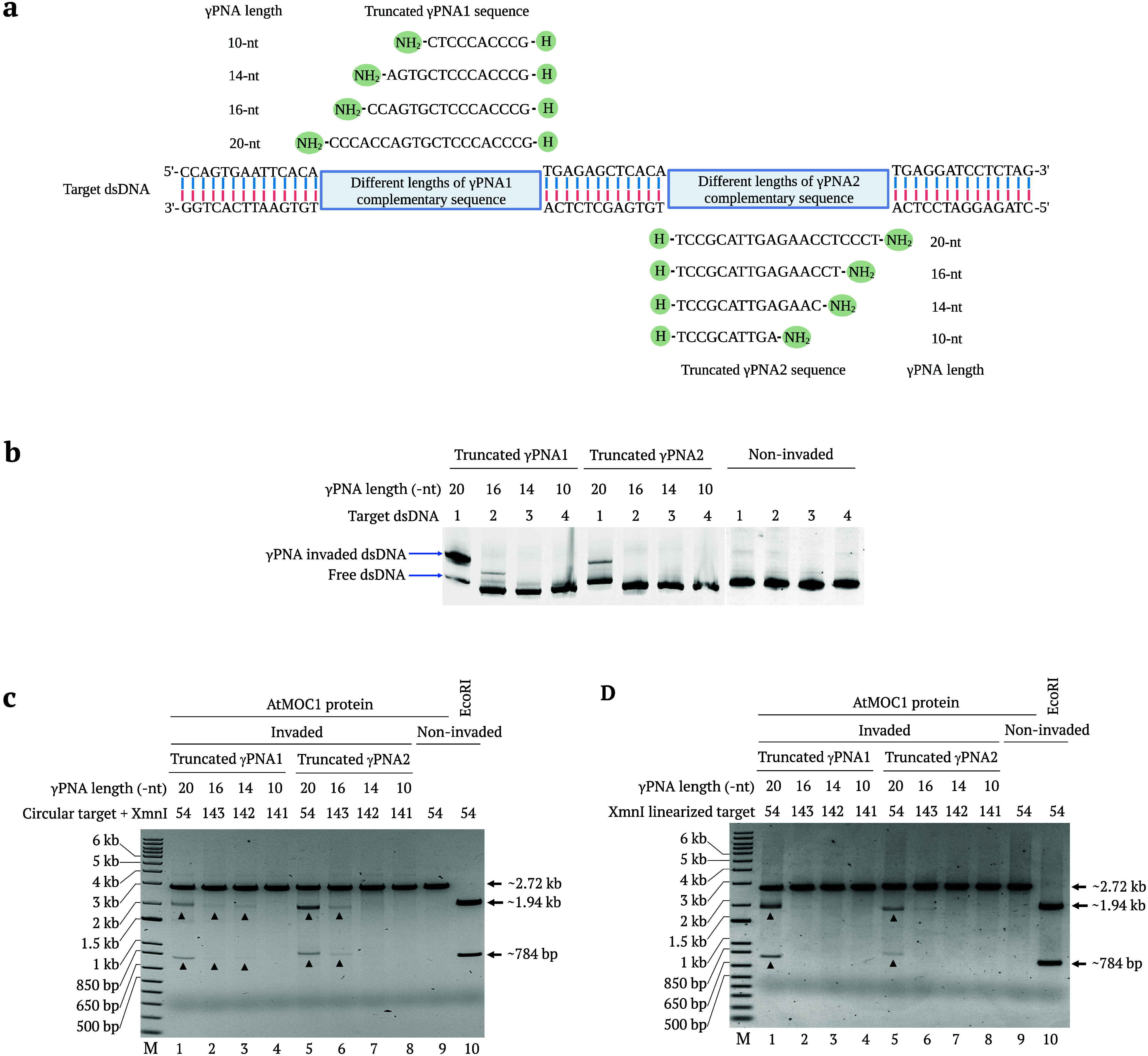
AtMOC1-mediated cleavage of dsDNA targets invaded by different lengths of γPNA molecules. (a) Sketch showing the dsDNA target-containing complementary sequences for the different truncated γPNA1 and γPNA2 molecules. (b) Gel mobility shift assay of the invasion of different truncated γPNAs into the corresponding dsDNA targets. (c, d) Gel images showing AtMOC1-mediated cleavage of different circular and XmnI-linearized plasmids invaded independently by different lengths of truncated γPNA molecules (Lanes 1–8). Lane 9 in both the gels is the AtMOC1-mediated cleavage of noninvaded circular and linear targets. XmnI was added in all of the circular plasmid cleavage reactions to release the fragment after AtMOC1-mediated DSB formation. Lane 10 in both gels is the restriction enzyme size control. Lane M shows the 1-kb plus DNA marker.

A recent study showed that 20-nt-long γPNA molecules are required for target cleavage using PNP editors.^16^ We examined different lengths of γPNAs to identify the proper length of PNA that causes efficient invasion and better cleavage via AtMOC1. Different lengths (10, 14, 16, and 20 nt) of γPNA1 and γPNA2 sequences and the Tm values are listed in Table S3. We designed and cloned different pUC19 targets containing 10, 14, 16, and 20 nt γPNA1 and γPNA2 binding regions (Figure 5a). We initially tested the invasion efficiency of the truncated γPNAs by mobility shift assays. The PCR-amplified linear dsDNA templates containing 10, 14, 16, and 20 nt γPNA1 and γPNA2 binding regions were incubated with the corresponding length γPNA molecules at 37 °C for 4 h. The invaded and noninvaded templates were separated by 6%TBE gel electrophoresis. Both 20 and 16 nt γPNA1 showed clear invasion into the corresponding dsDNA target, whereas only 20 nt γPNA2 showed invasion into the corresponding dsDNA target (Figure 5b). We confirmed the efficiency of invasion of different lengths of γPNA molecules by performing AtMOC1-mediated cleavage by invading different circular and XmnI-linearized pUC19 targets with γPNA molecules of the corresponding lengths. Following AtMOC1 treatment, we observed cleavage in targets invaded by 20-nt γPNA1 and γPNA2 in both the circular and the linear plasmids (Figure 5c,d). For the circular targets, we also detected cleavage in 16-nt γPNA1-invaded and 16-nt γPNA2-invaded targets (Figure 5c). The circular or linear targets invaded with 14 and 10 nt lengths of PNAs did not display any cleavage after AtMOC1 treatment. Consistent with the previous data, the current results also show that 20-nt γPNA was best able to invade linear targets and showed clear target cleavage with AtMOC1. We also observed the invasion of 16-nt-long γPNA molecules into circular targets and precise cleavage with AtMOC1. The promising AtMOC1-mediated cleavage activity with shorter PNAs increases the design possibilities and chances for delivery, facilitating the use of PNR in vivo.

### Measuring AtMOC1 Activity at Different Temperatures, Time Points, and Reaction Buffer Conditions

**Figure 6:**
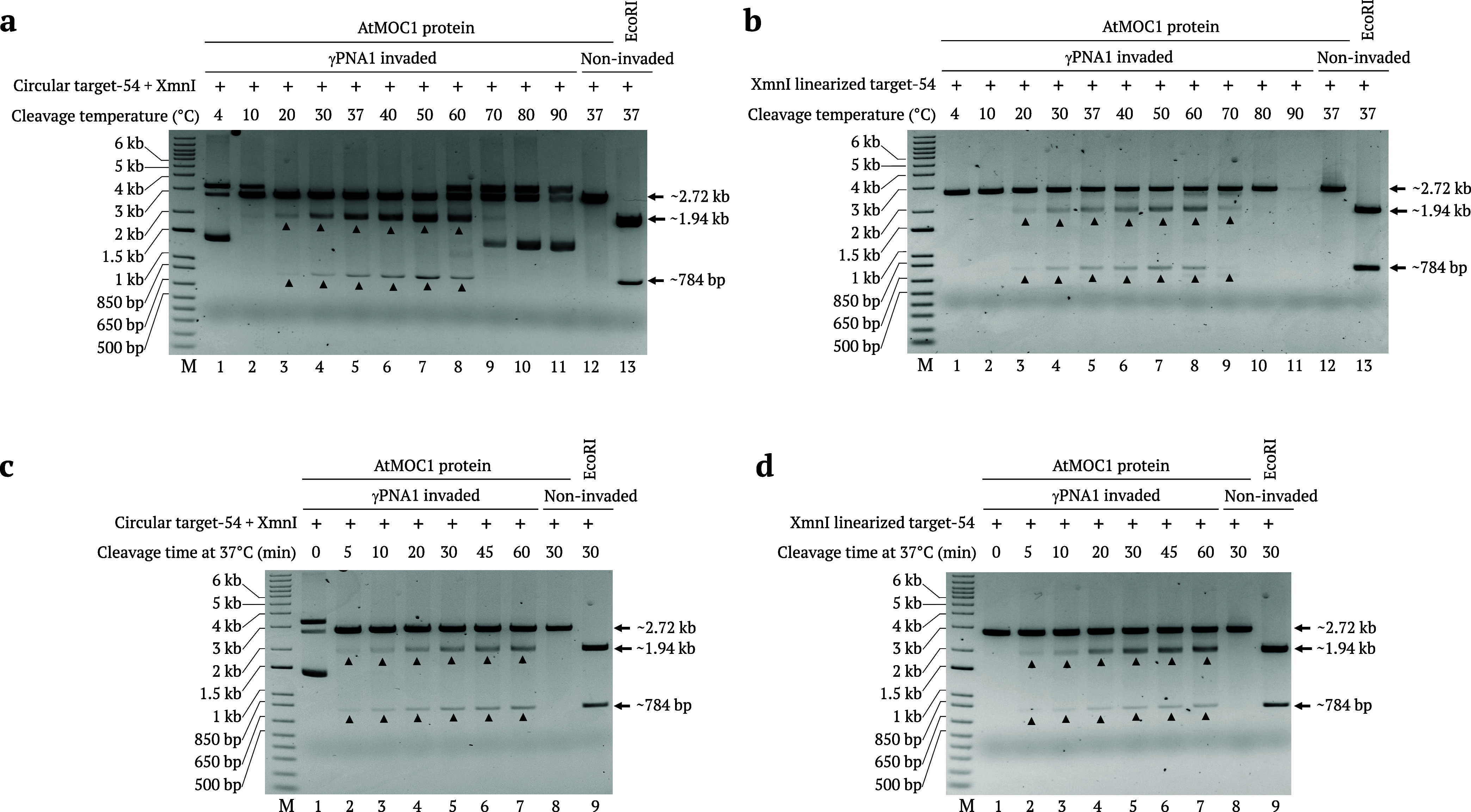
AtMOC1 activity assay using the γPNA1-invaded target at different temperatures and time points. (a, b) Gel images showing the activity of AtMOC1 on γPNA1-invaded circular and XmnI-linearized pUC19 target-54 at temperatures ranging from 4 to 90 °C (Lanes 1–11). Lane 12 in (a) and (b) gels showing AtMOC1-mediated cleavage of noninvaded circular and XmnI-linearized targets at 37 °C. XmnI was added to all of the circular plasmid cleavage reactions to release a fragment after AtMOC1-mediated cleavage. Restriction enzyme size control included in lane 13 in (a) and (b) gels. (c, d) Gel images showing AtMOC1 activity on γPNA1-invaded circular and XmnI-linearized pUC19 target-54 at different time points, independently (Lanes 1–7). Lane 8 in (c) and (d) gels showing AtMOC1-mediated cleavage of noninvaded circular and XmnI-linearized targets after 30 min. XmnI was added to all of the circular plasmid cleavage reactions to release a fragment after AtMOC1-mediated cleavage. Restriction enzyme size control included in lane 9 in (c) and (d) gels. Lane M shows the 1-kb plus DNA marker.

Optimizing the in vitro invasion and cleavage conditions of γPNA and AtMOC1, respectively, is important for future in vitro site-specific cleavage and cloning experiments. γPNA molecules can invade dsDNAs at temperatures greater than 25 °C.^16^ Here we assessed the activity of AtMOC1 at different temperatures. We first invaded the circular or XmnI-linearized target-54 with γPNA1 at 37 °C. The invaded targets were subjected to AtMOC1-mediated cleavage at 4, 10, 20, 30, 37, 40, 50, 60, 70, 80, and 90 °C. AtMOC1 cleaved the PNA-invaded targets at temperatures ranging from 20 to 60 °C. We observed less AtMOC1-mediated cleavage at 70 °C, which occurred in XmnI-linearized targets. AtMOC1-mediated cleavage of circular plasmids also requires the collective activity of XmnI. This restriction enzyme might be inactive at temperatures greater than 70 °C, perhaps explaining the lack of a cleavage fragment from circular template at 70 °C. No AtMOC1-mediated cleavage was observed at temperatures greater than 70 °C. We determined that 37 °C would be the ideal temperature for in vitro cleavage experiments without any nonspecific cleavage (Figure 6a,b). The successful cleavage activity of AtMOC1 at physiological temperatures supports its utilization for in vivo genome editing applications.

We evaluated the AtMOC1 activity at different time points. Circular or XmnI-linearized target-54 was invaded by γPNA1, and the invaded templates were subjected to AtMOC1-mediated cleavage via incubation for 0, 5, 10, 20, 30, 45, or 60 min at 37 °C. AtMOC1 cleaved both the circular and linear targets after 5 min of incubation at 37 °C. We observed a gradual increase in cleavage band intensity with increasing duration of AtMOC1-mediated cleavage (Figure 6c,d). Prolonging the AtMOC1 cleavage time for 1 h did not have any negative effect on overall PNR editor-mediated DSB formation. Time course experiments revealed the robustness of AtMOC1 for cleaving the γPNA-invaded target.

We also tested the γPNA invasion at different time points. We invaded circular and XmnI-linearized pUC19-target-54 with γPNA1 and incubated the reactions at 37 °C for 0, 5, 15, 30, 45, 60, 90 min and 2, 4, 6, 8, and 16 h. We then subjected the reactions to AtMOC1-mediated cleavage at 37 °C for 30 min. Cleavage results showed that γPNA1 invaded the circular target immediately after its addition (Figure S5a), whereas γPNA1 invaded the linear target after a 5 min incubation at 37 °C (Figure S5b). In both cases, the γPNA-invaded template was stable and was cleaved by AtMOC1 even after 16 h of γPNA invasion. In all of these cleavage experiments, we incubated γPNA1 with the circular target for 1 h and with the linear target for 6 h at 37 °C.

Investigating the optimal buffer conditions for γPNA invasion and AtMOC1 activity is important for in vitro and in vivo applications of the PNR tool. Earlier studies showed successful γPNA invasion in MOPS buffer.^15,16^ We therefore invaded XmnI-linearized pUC19-target-54 with γPNA1 in a MOPS buffer and subjected the invaded target to AtMOC1-mediated cleavage in no buffer, NEB-rCutsmart, NEB-r1.1, NEB-r2.1, NEB-r3.1, HEPES, MOPS, or PBS buffer. AtMOC1 successfully cleaved the target DNA in no buffer, NEB-rCutsmart, NEB-r1.1, NEB-r2.1, NEB-r3.1, HEPES, and PBS buffer, whereas no cleavage was observed in MOPS buffer (Figure S6a). We further validated the invasion of γPNA in different buffers by conducting γPNA1 invasion into circular and linear targets in no buffer, NEB-rCutsmart, NEB-r1.1, NEB-r2.1, NEB-r3.1, HEPES, MOPS, and PBS buffers. We then subjected the γPNA1-invaded circular and linear targets to AtMOC1-mediated cleavage in NEB-rCutsmart buffer. γPNA1 successfully invaded the circular plasmids in no buffer, NEB-rCutsmart, NEB-r1.1, NEB-r2.1, NEB-r3.1, MOPS, and PBS buffers. However, no invasion occurred in the HEPES buffer (Figure S6b). In the case of γPNA1 invasion into linear DNA and AtMOC1-mediated cleavage, γPNA1 invaded the target only in no buffer and MOPS buffer. We did not observe any γPNA1 invasion in the presence of NEB-rCutsmart, NEB-r1.1, NEB-r2.1, NEB-r3.1, HEPES, or PBS buffer (Figure S6c). We also tested the γPNA1 invasion and AtMOC1-mediated cleavage simultaneously in no buffer, NEB-rCutsmart, NEB-r1.1, NEB-r2.1, NEB-r3.1, HEPES, MOPS, or PBS buffer. γPNA1 invasion and AtMOC1-mediated cleavage occurred only in no buffer: no invasion or cleavage was observed in any of the buffers tested (Figure S6d). These results indicate that identifying a PNA molecule that is functional in different buffer conditions for efficient invasion is indispensable for the successful use of PNR editors in vivo.

### Titration Assay of AtMOC1 and γPNA1 Concentrations

Resolvases are a class of endonucleases that possess nonspecific target DNA chopping activity at higher protein concentrations in vitro. Identifying the correct AtMOC1 protein concentration for in vitro cleavage experiments is essential for downstream PNR applications. To assess the activity of different concentrations of AtMOC1 protein, we performed a cleavage experiment with different AtMOC1 protein concentrations. We invaded circular and XmnI-linearized pUC19 target-54 with γPNA1 and subjected the invaded and noninvaded circular and linear plasmids to AtMOC1-mediated cleavage with different concentrations (0–1 μM) of AtMOC1. AtMOC1 at concentrations of 50–250 nM showed very precise cleavage of γPNA1-invaded circular and linear targets. However, AtMOC1 at concentrations >250 nM showed nonspecific chopping of invaded and noninvaded targets (Figure S7a,b).

Similarly, identifying the proper γPNA concentration for sufficient target invasion for AtMOC1-mediated cleavage is crucial for further PNR editing applications. We therefore invaded the XmnI-linearized pUC19 target-54 with different concentrations (0–4 μM) of γPNA1 or γPNA2. The γPNA-invaded targets were subjected to AtMOC1-mediated cleavage at 37 °C for 30 min. PNA invasion took place at >50 nM concentrations of γPNA1 or γPNA2 (Figure S8a,b). Higher concentrations of γPNA molecules did not have any negative effects on the cleavage activity of AtMOC1.

### γPNA Target Mismatch Specificity Assay via AtMOC1-Mediated Cleavage

Validating γPNA specificity toward the correct targets is crucial for demonstrating the precision of the PNR editing concept. For this experiment, we designed different targets containing different numbers of mismatches at the 5′ end, 3′ end, and central region and nonconsecutive mismatches in the targets that bind with γPNA1 molecules (Figure S9a,b). All mismatched target oligos were reannealed and separately cloned into the pMRS vector. To test the target specificity of γPNA1 via AtMOC1-mediated cleavage, we invaded the BsrGI-linearized mismatched pMRS targets with γPNA1 and performed AtMOC1-mediated cleavage at 37 °C for 30 min. Cleavage results showed that γPNA1 invaded the targets containing 1-4-nt mismatches at the 5′ ends and a 1-nt mismatch at the 3′ ends of the targets. Conversely, targets with 5-11-nt mismatches at the 5′ end, 2-11-nt mismatches at the 3′ end, all central mismatches, and all nonconsecutive mismatches did not show the γPNA1 invasion, as evidenced by the lack of AtMOC1-mediated cleavage (Figure S9c). This brings more value to the specificity of PNR editors toward the target dsDNA.

### AtMOC1-Mediated Multiplex Cleavage of Circular and Linear Targets

**Figure 7:**
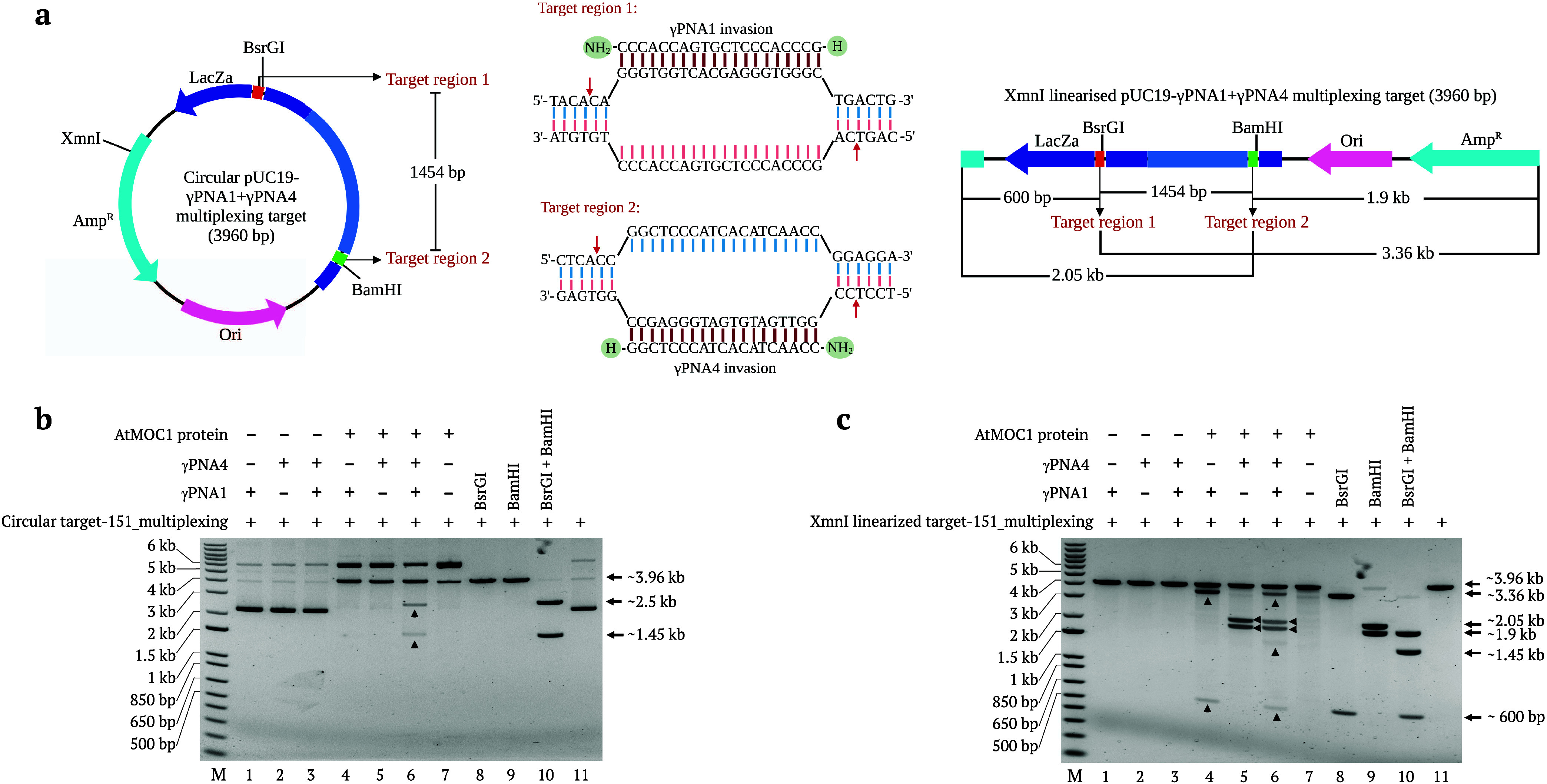
AtMOC1-mediated multiplex cleavage of γPNA-invaded circular and linear plasmids. (a) Circular and XmnI-linearized pUC19 multiplex target-151 containing γPNA1 and γPNA4 binding regions. Restriction sites and the fragments released after multiplex cleavage are indicated in the plasmid maps. Target regions 1 and 2 are the sequences that can be invaded by γPNA1 and γPNA4 molecules, respectively. AtMOC1 cleavage sites are indicated by arrows. (b, c) Gel images showing the AtMOC1-mediated multiplex cleavage of circular and XmnI-linearized plasmids, respectively. Lanes 1–3 in both gels are the only invasion of targets with γPNA1, γPNA4, and γPNA1+γPNA4, respectively. Lanes 4–6 in both gels are the AtMOC1-mediated cleavage of γPNA1, γPNA4, and γPNA1+γPNA4 invaded targets, respectively. Lane 7 in both gels shows the AtMOC1-mediated cleavage of noninvaded targets. Lanes 8–11 in both the gels are restriction digestions as the size controls for AtMOC1-mediated multiplex cleavage. Lane M shows the 1-kb plus DNA marker.

Multiplex γPNA invasion and multiple site cleavage using AtMOC1 are required for the precise fragment release. Multiplex target cleavage can unlock the potential of PNR editors for in vitro cloning of larger genomic fragments and in vivo fragment deletion. To evaluate the efficiency of PNR-mediated multiplex cleavage, we cloned pUC19 multiplexing target-151 containing the γPNA1 and γPNA4 binding regions with a 1454 bp spacer, as described in the Methods. We then invaded the circular and XmnI-linearized multiplexing target-151 plasmid with γPNA1, γPNA4 or γPNA1+γPNA4. γPNA1 designed to invade the top strand at target region-1, and γPNA4 designed to invade the bottom strand at target region-2 (Figure 7a). The invaded and noninvaded targets were subjected to AtMOC1-mediated cleavage at 37 °C for 30 min. Circular plasmids invaded by both γPNA1 and γPNA4 had undergone AtMOC1-mediated multiplex cleavage and released fragments of the expected sizes. By contrast, the PNA-invaded targets without AtMOC1, single PNA-invaded circular targets, and noninvaded circular targets did not show any fragment release (Figure 7b). Similarly, XmnI-linearized plasmids invaded by both γPNA1 and γPNA4 showed AtMOC1 cleavage bands of the expected sizes corresponding to multiplex cleavage. Whereas, the linear plasmid invaded by γPNA1 or γPNA4 showed AtMOC1-mediated cleavage and fragment release corresponding to single PNA invasion. Conversely, PNA-invaded linear targets without AtMOC1 and noninvaded linear targets with AtMOC1 did not show any fragment release (Figure 7b). Multiplexing with PNR editors is much simpler than with other methods as it requires invasion of only two PNA molecules and AtMOC1.

## Conclusions

In this study, we demonstrated the AtMOC1-mediated cleavage of γPNA invaded the dsDNA substrate very precisely. We evaluated the nucleotide requirement for AtMOC1-mediated cleavage and showed that AtMOC1 can cleave any PNA-invaded substrate without the nucleotide specificity. We determined the AtMOC1 cleavage sites of the γPNA-invaded substrates. We tested different lengths of γPNA molecules and observed better cleavage with 20-nt-long γPNA molecules. Our multiplex cleavage assay demonstrates the precise release of a dsDNA fragment that can be used for further cloning applications. Overall, we harnessed the eukaryotic AtMOC1 protein and γPNA molecule to produce site-specific DSBs. More research on the structural characteristics of binding of AtMOC1 protein to γPNA-invaded targets could reveal interesting information about the protein recognition and cleavage of γPNA-invaded DNA structures resembling HJs. In the future, resolvases could play a vital role in in vivo genome engineering, in vitro sequence-independent cloning of larger genomic fragments, and genome assembly. PNR editors have several advantages over other site-specific nucleases, including the PAM-independent target recognition, their strong activity under different physiological conditions, the simple-to-design reactions that require few PNR reagents, and the smaller size of the reagents which can facilitate the easy delivery of these editors in vivo.

## Data Availability

The data from this article are available in the article and the Supporting Information.

## Supporting Information Available

Supporting methods; mobility shift assay (Figure S1); AtMOC1 cleavage of different targets containing different nucleotide compositions around the γPNA invasion (Figures S2 and S3); AtMOC1 cleavage of different γPNA invaded targets (Figures S4); γPNA1 invasion at different time points and AtMOC1 cleavage (Figure S5); AtMOC1 activity assay in different buffers (Figure S6); AtMOC1 protein concentration titration assay (Figure S7); γPNA concentration titration assay (Figure S8); AtMOC1 cleavage of γPNA1 invaded mismatch targets (Figure S8); protein expression vectors used in this study; target plasmids used in this study; primers used in this study (Table S1); AtMOC1 protein sequence (Table S2); different PNA sequences tested in this study (Table S3); and top and bottom oligos used to clone the target sequences in pUC19 or pMRS vectors (Table S4) (PDF)
