# Supplementary material for: Harnessing Peptide Nucleic Acids and the Eukaryotic Resolvase MOC1 for Programmable, Precise Generation of Double-Strand DNA Breaks
Source: Anal Chem. 2024 Feb 1;96(6):2599–609. doi: 10.1021/acs.analchem.3c05133 (PMC10867802; doi:10.1021/acs.analchem.3c05133)
Supplement: Supplementary file 1 — ac3c05133_si_001.pdf [file ac3c05133_si_001.pdf]

Supporting information

Harnessing peptide nucleic acids and the eukaryotic resolvase MOC1 for programmable, precise generation of double-strand DNA breaks

Gundra Sivakrishna Rao[1], Ahmed H. Saleh[1], Firdaws Melliti[1], Syed Muntjeeb[1], and Magdy Mahfouz[1*]

[1]*Laboratory for Genome Engineering and Synthetic Biology, Division of Biological Sciences, 4700 King Abdullah University of Science and Technology, Thuwal 23955-6900, Saudi Arabia.*

***Correspondence: Magdy M. Mahfouz (magdy.mahfouz@kaust.edu.sa)**

Supplementary methods

AtMOC1 protein purification

The pCold I_ AtMOC1 plasmid was transformed into *E. coli* BL21DE3 cells. A single colony was inoculated into 100 ml of Luria-Bertani (LB) medium supplemented with 100 mg/ml ampicillin and cultured at 37°C overnight. A 20 ml aliquot of the overnight grown culture was inoculated into 1 lit TB (Terrific Broth from IBI Scientific - IB49140) broth medium (50.8 gm TB medium + 4ml of glycerol into 1 lit distilled water) supplemented with 100 mg/ml ampicillin; four independent 1 lit flasks were prepared. The cultures were incubated in a shaker incubator until the OD-600 reached to 0.4. The flasks were incubated at 4°C for 20-30 minutes. Protein expression in cells was induced by adding 0.5 mM IPTG and incubation at 16°C in a shaker incubator for 18 hrs. The induced *E. coli* culture was centrifuged at 6000rpm for 15 mins at 4°C. The pellet was resuspended and lysed in 100 ml lysis buffer (50mM Tris HCL pH 7.5, 300mM NaCl, 20mM imidazole, 5% glycerol, Protease inhibitor-2 tablets for 100 ml (Thermo Scientific, A32953), 1mM TCEP HCl, 2 mg/1 ml Lysozyme (Sigma Aldrich, L6876), 10% NP40, 50 µl of β-Mercaptoethanol, and 1 µl/100 ml Benzonase® Nuclease (Merck, E1014-5KU)) for 45 mins at 4°C. The cells were also lysed by sonication (Qsonica Q700). The lysed cells were centrifuged at 16,000 rpm for 45 min at 4°C (Eppendorf, 5810R), and the supernatant containing 6xHis-AtMOC1 was collected and filtered through a 0.45µm bottletop vacuum filter (Thermo Scientific™ - 2914545).

The supernatant containing 6xHis-AtMOC1 was applied onto a Ni-NTA column (HisTrap HP, 5 mL GE Healthcare), and affinity column chromatography was performed using the ÄKTA Pure system (GE Healthcare) to capture the 6xHis tagged AtMOC1 protein. The unbound proteins were removed by washing with buffer A (50mM Tris-HCl pH 7.5, 500mM NaCl, 20mM imidazole, 1mM TCEP), and 6xHis tagged AtMOC1 was eluted with buffer B (50mM Tris-HCl pH 7.5, 500mM NaCl, 300mM imidazole and 1mM TCEP). The protein fractions were collected in a SnakeSkin™ Dialysis membrane (Thermo Scientific™ - 68100) and subjected to overnight dialysis in dialysis buffer (50mM Tris-HCl pH 7.5, 100mM NaCl, and 5% [v/v] glycerol). The dialyzed AtMOC1 protein was applied to an ion exchange column (HiTrap™ Heparin HP, 5 mL GE Healthcare), and ion exchange chromatography was performed using the ÄKTA Pure system (GE Healthcare). The column was washed with low salt buffer (50mM Tris-HCl pH 7.5, 100mM NaCl, 1mM TCEP), and AtMOC1 protein was eluted with high-salt buffer (50mM Tris-HCl pH

7.5, 2M NaCl, 1mM TCEP). The protein fractions were buffer exchanged three times with low salt buffer and subjected to size exclusion chromatography by filtering through an S200 column (GE Healthcare) in size exclusion chromatography buffer (25mM Tris-HCl pH 7.5, 100mM NaCl, 1mM TCEP and 10% [v/v] glycerol). The AtMOC1 protein fractions were combined, the concentration was adjusted to 10 μM, and the aliquots were snap-frozen in liquid nitrogen and stored at -80°C for further experiments. The protein sequences are listed in Table S2.

Supplementary figures

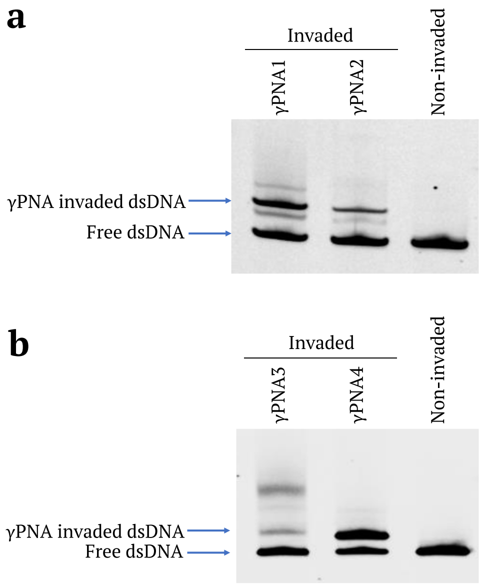

Figure S1. Mobility shift assay of different γPNA molecules invading into corresponding dsDNA target. **(a)** and **(b)** 6%TBE gels showing the dsDNA targets invaded with γPNA1, γPNA2, γPNA3 and γPNA4, independently. Non-invaded dsDNA template also ran in the same gel for the size reference.

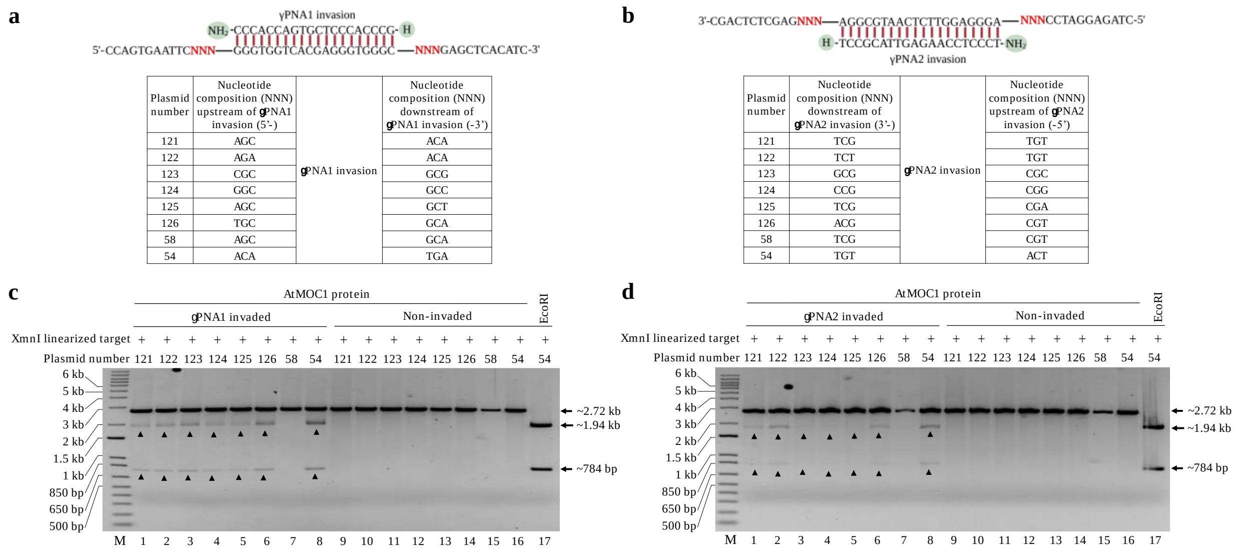

Figure S2. AtMOC1 cleavage of different XmnI linearized targets containing different nucleotide composition around the γPNA1 and γPNA2 invasions, independently. (a) and **(b)** Tables showing the nucleotide composition at the both ends of γPNA1 and γPNA2 invasion in different pUC19 target plasmids. In the sketch, NN represents the nucleotides that are replaced with different nucleotides in different targets, rest of the pUC19 plasmid sequence is same in all the targets. **(c)** and **(d)** Gel pictures showing the AtMOC1 cleavage of γPNA1 and γPNA2 invaded XmnI linearized dsDNA, respectively. Lanes 1-6 and 8 in both the gels are the different XmnI linearized targets invaded separately with γPNA1 and γPNA2, showing the AtMOC1 cleavage and a fragment release at the corresponding sizes. Conversely, the lane 7 in both the gels is the pUC19-58 target invaded separately with γPNA1 and γPNA2, did not show any cleavage with AtMOC1. Whereas, lanes 9-16 in both the gels are non-invaded XmnI linearized targets, did not show any cleavage after AtMOC1 cleavage. Lane 17 is the XmnI linearized pUC19-54 target treated with EcoRI restriction enzyme is a size control for the AtMOC1 cleavage reactions. Lane M represents the 1-kb plus DNA marker.

a

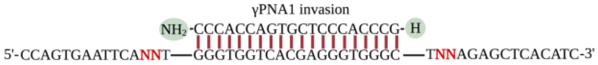

Plasmid number	Nucleotide composition (NN) upstream of γPNA1 invasion (5'-)		Nucleotide composition (NN) downstream of γPNA1 invasion (-3')
144	CA	γPNA1 invasion	TG
145	CC		GG
146	CG		CG
147	CT		AG
148	AC		GT
149	GC		GC

b

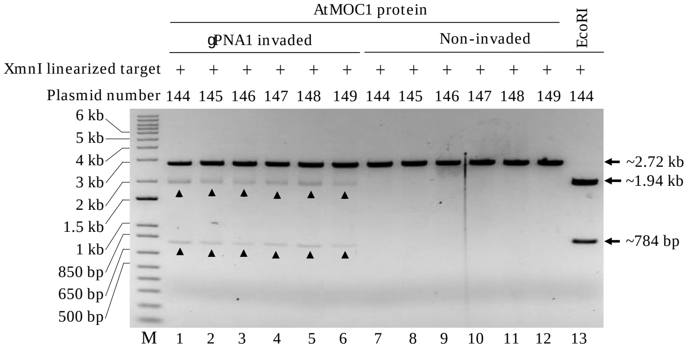

Figure S3. AtMOC1 cleavage of different XmnI linearized targets containing different nucleotide composition around the γPNA1 invasions, independently. (a) Table showing the nucleotide composition at the both ends of γPNA1 invasion in different pUC19 target plasmids. In the sketch, NN represents the nucleotides that are replaced with different nucleotides in different targets, rest of the pUC19 plasmid sequence is same in all the targets. **(b)** Gel picture showing the AtMOC1 cleavage of γPNA1 invaded XmnI linearized dsDNA. Lanes 1-6 are the different XmnI linearized targets invaded separately with γPNA1, showing the AtMOC1 cleavage and a fragment release at the corresponding sizes. Whereas, lanes 7-12 are non-invaded XmnI linearized targets, did not show any cleavage after AtMOC1 cleavage. Lane 13 is the XmnI linearized pUC19-144 target treated with EcoRI restriction enzyme is a size control for the AtMOC1 cleavage reactions. Lane M represents the 1-kb plus DNA marker.

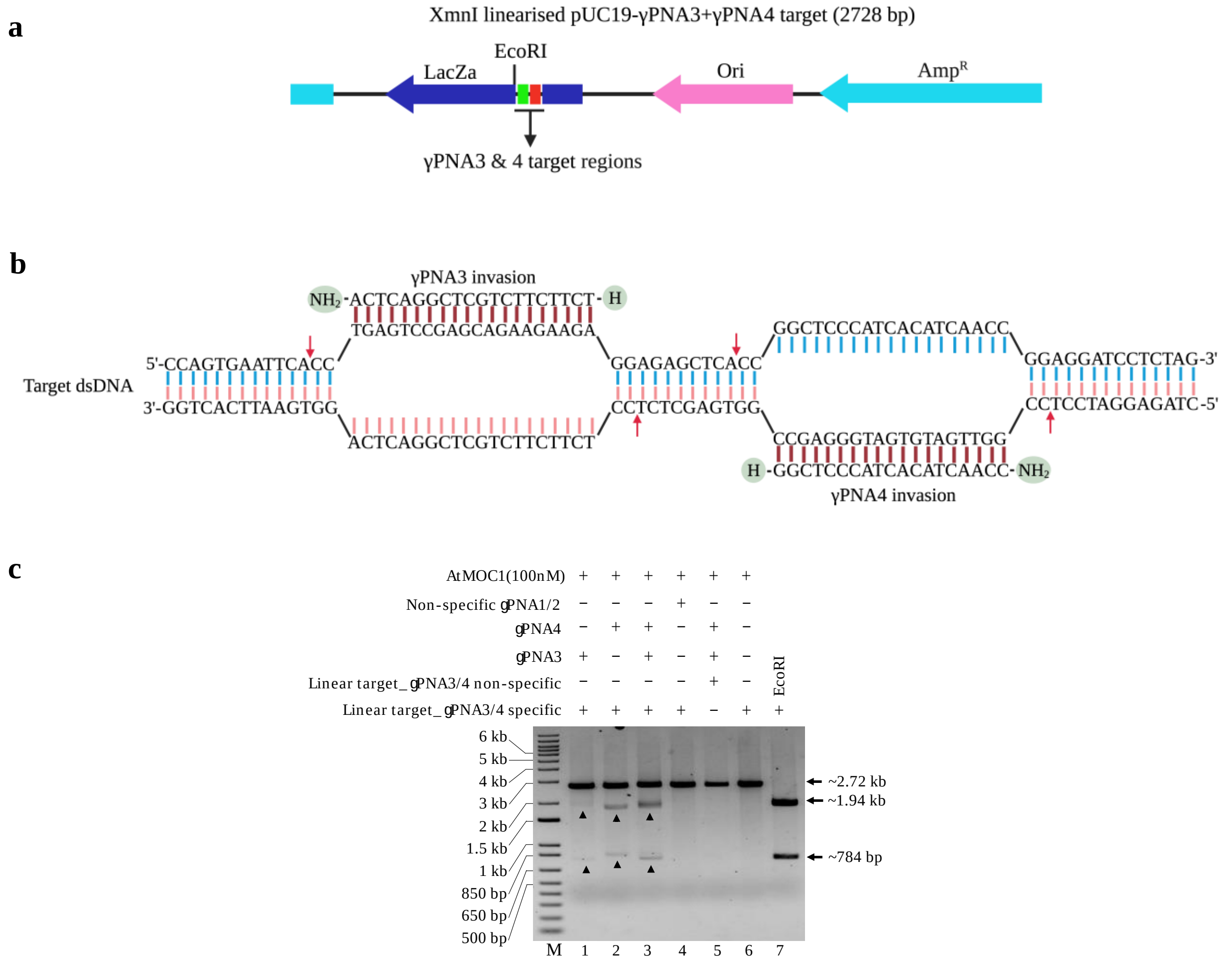

Figure S4. AtMOC1 cleavage of γPNA3 or γPNA4 invaded XmnI linearized pUC19-49 plasmid. (a) XmnI linearized pUC19-49 map showing γPNA3 and γPNA4 binding regions and restriction enzyme sites. **(b)** Sketch representing the γPNA binding regions 1 and 2 in pUC19-49 plasmid. γPNA3 invades the top strand and γPNA4 invades the bottom strand. Arrow marks indicate the positions of AtMOC1 cleavage sites. **(c)** Gel image showing the AtMOC1 cleavage of γPNA3 or γPNA4 invaded XmnI linearized dsDNA. Lanes 1-3 showing the release of AtMOC1 cleavage fragments at the expected sizes upon cleavage of the XmnI linearized pUC19-49 target invaded with γPNA3, γPNA4 and γPNA3+γPNA4, respectively. Lanes 4-6 showing no AtMOC1 cleavage products in the non-specific γPNA1+γPNA2 invaded XmnI linearized plasmid, non-specific XmnI linearized pUC19-54 target invaded with γPNA3+γPNA4 and non-invaded XmnI linearized pUC19-49 targets treated with AtMOC1, respectively. Lanes 7 is the XmnI linearized pUC19-49 plasmid treated with EcoRI. This is a size control reaction for the AtMOC1 cleavage reactions. Lane M represents the 1-kb plus DNA marker.

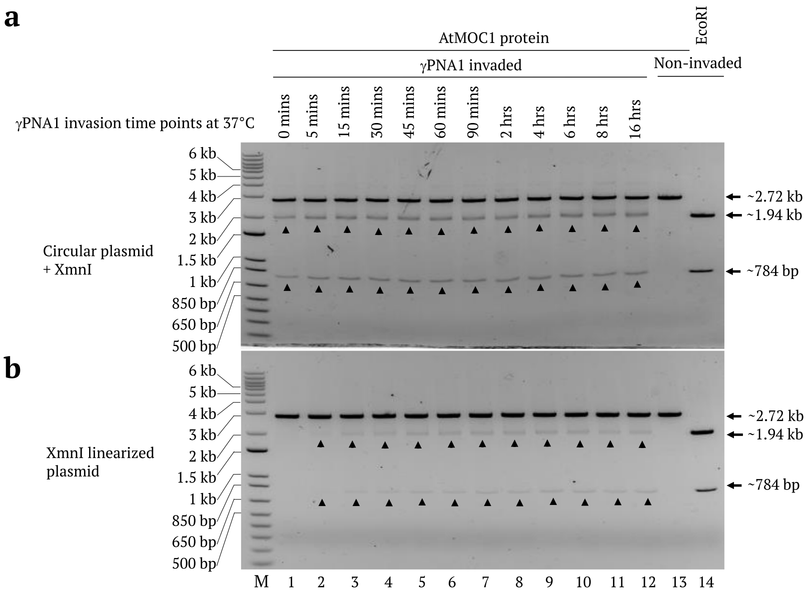

Figure S5. γPNA1 invasion into circular and linearized targets at different time points. (a) Gel image showing the γPNA1 invasion into circular pUC19-54 plasmid at different time points. Lanes 1-12 are the γPNA1 invasion into circular pUC19-54 template at 37°C for 0-16 hrs, respectively. γPNA1 invasion observed immediately after adding PNA to the circular template, evidenced by AtMOC1 cleavage. Lane 13 is the AtMOC1 cleavage of non-invaded circular target showing no fragment release. In all the above reactions, XmnI restriction enzyme added in the cleavage reaction to release a fragment together with AtMOC1 cleavage. Lane 14 is the circular plasmid treated with EcoRI+XmnI restriction enzymes, is the size control for the AtMOC1 cleavage reactions. **(b)** Gel image showing the γPNA1 invasion into XmnI linearized pUC19-54 plasmid at different time points. Lanes 1-12 are the γPNA1 invasion into linear pUC19-54 template at 37°C for 0-16 hrs, respectively. γPNA1 invasion observed 5 mins after incubating template with γPNA1, evidenced by the AtMOC1 cleavage. Lane 13 is the AtMOC1 cleavage of non-invaded linear target showing no fragment release. Lane 14 is the linear plasmid treated with EcoRI restriction enzyme, is the size control for the AtMOC1 cleavage reactions. Lane M represents the 1-kb plus DNA marker.

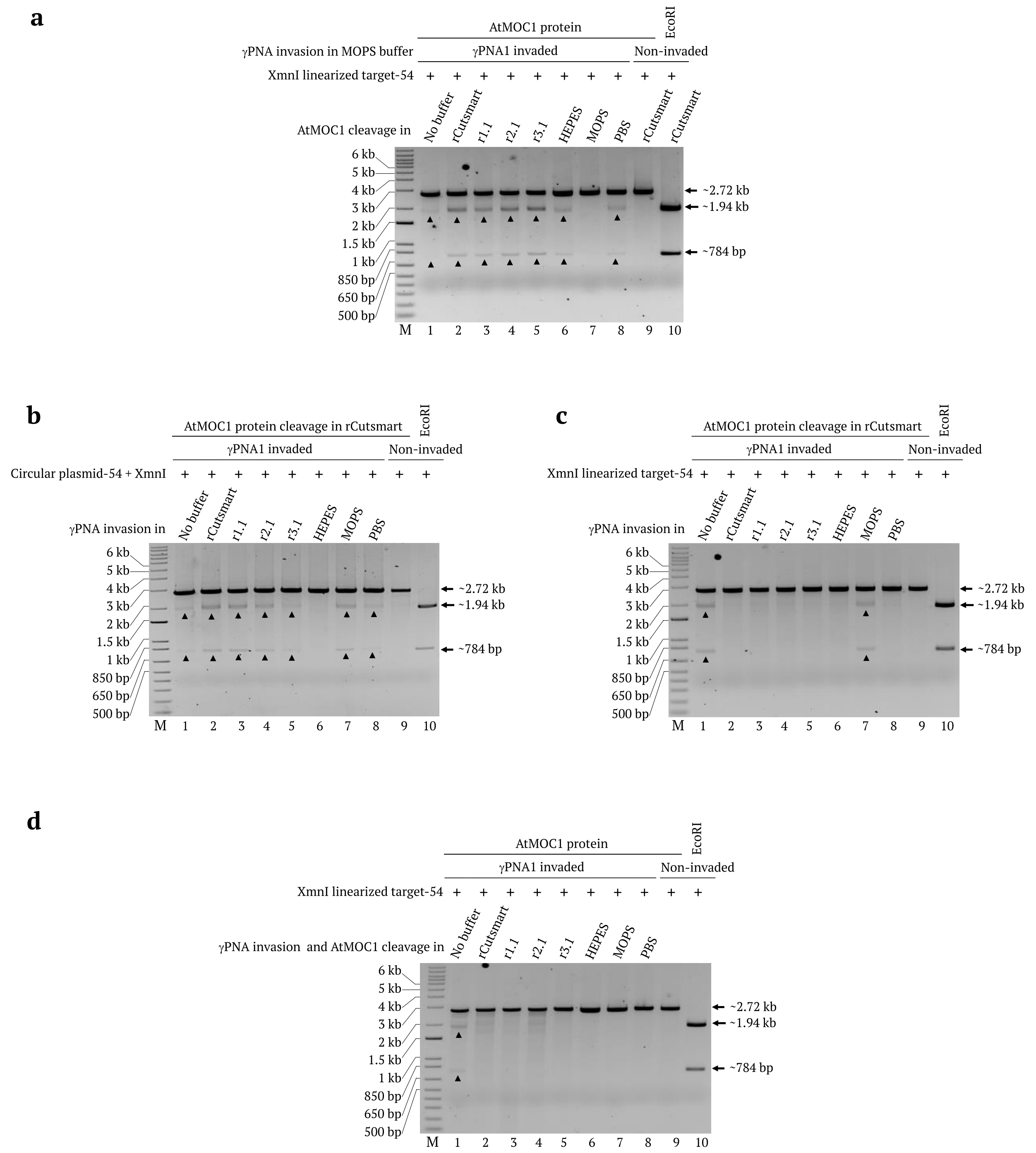

Figure S6. AtMOC1 activity assay using γPNA1-invaded targets in different buffers. (a) Gel image showing γPNA1 invasion into XmnI-linearized pUC19 target-54 plasmid in 1X MOPS buffer and the AtMOC1 cleavage reaction conducted in no buffer, NEB-rCutsmart, NEB-r1.1, NEB-r2.1, NEB-r3.1, HEPES, MOPS, PBS buffers (Lanes 1-8, respectively) at 37°C for 30 mins. AtMOC1-mediated cleavage was observed using all buffers except MOPS (Lane 7). Lane 9 shows the AtMOC1-mediated cleavage of non-invaded linear target in NEB-rCutsmart showing no fragment release. Lane 10 is the linear plasmid treated with EcoRI, which served as the size control for the

AtMOC1 cleavage reactions. (b) Gel image showing γPNA1 invasion into circular pUC19 target-54 in no buffer, NEB-rCutsmart, NEB-r1.1, NEB-r2.1, NEB-r3.1, HEPES, MOPS, PBS buffers (Lanes 1-8, respectively) and AtMOC1-mediated cleavage in NEB-rCutsmart buffer for 30 mins at 37°C. γPNA1 invasion into circular plasmids was observed using all buffers except HEPES (Lane 6), as evidenced by AtMOC1 cleavage. Lane 9 shows AtMOC1-mediated cleavage of non-invaded circular target in NEB-rCutsmart showing no fragment release. In all these circular plasmid cleavage reactions, XmnI was added to release a fragment together with AtMOC1 cleavage. Lane 10 is the circular plasmid treated with EcoRI+XmnI as the size control for the AtMOC1 cleavage reactions. (c) Gel image showing γPNA1 invasion into XmnI-linearized pUC19 target-54 in no buffer, NEB-rCutsmart, NEB-r1.1, NEB-r2.1, NEB-r3.1, HEPES, MOPS, PBS buffers (Lanes 1-8, respectively) and AtMOC1-mediated cleavage in NEB-rCutsmart buffer for 30 mins at 37°C. γPNA1 invasion into linearized plasmids was observed in no buffer and MOPS buffer (Lane 1 and 7), as evidenced by AtMOC1-mediated cleavage in NEB-rCutsmart buffer. Lane 9 shows AtMOC1-mediated cleavage of the non-invaded linear target in NEB-rCutsmart buffer showing no fragment release. Lane 10 shows the linear plasmid treated with EcoRI as the size control for the AtMOC1 cleavage reactions. (d) Gel image showing γPNA1 invasion into XmnI-linearized pUC19 target-54 and AtMOC1-mediated cleavage in no buffer, NEB-rCutsmart, NEB-r1.1, NEB-r2.1, NEB-r3.1, HEPES, MOPS, and PBS buffers (Lanes 1-8, respectively) for 30 mins at 37°C. γPNA1 invasion and AtMOC1 cleavage were observed in no buffer only (Lane 1). Lane 9 shows AtMOC1-mediated cleavage of the non-invaded linear target in NEB-rCutsmart buffer showing no fragment release. Lane 10 is the linear plasmid treated with EcoRI as the size control for the AtMOC1 cleavage reactions. Lane M shows the 1-kb plus DNA marker.

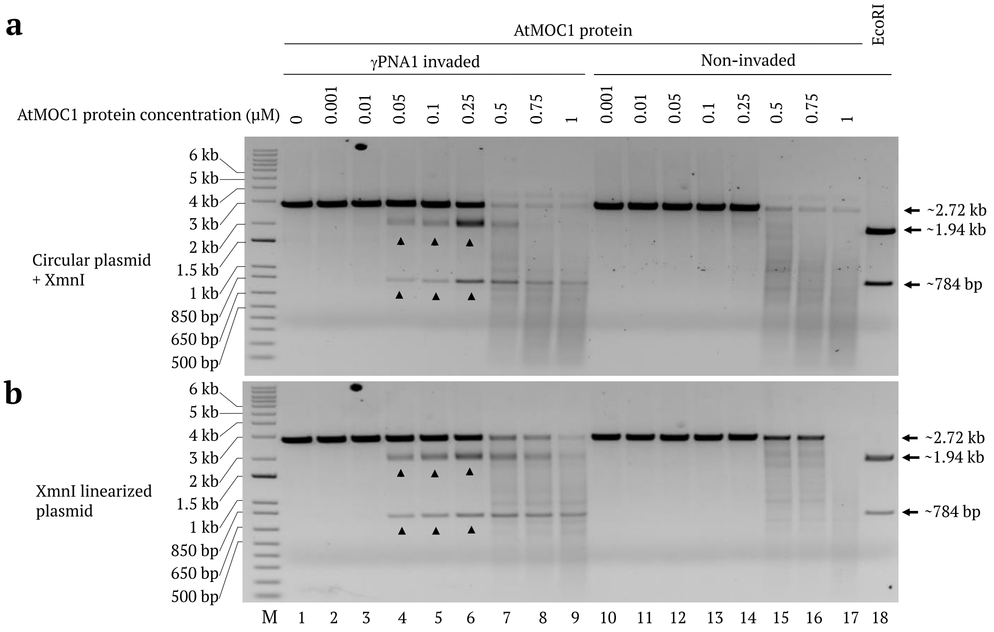

Figure S7. AtMOC1 protein concentration titration assay on circular and linear pUC19-54. (a) and **(b)** Gel images showing γPNA1 invaded and non-invaded circular and XmnI linearized plasmids, respectively treated with different concentrations of AtMOC1. Lanes 1-9 are the γPNA1 invaded targets treated with 0 to 1µM AtMOC1 protein, respectively. Lanes 1-3 did not show any cleavage. Lanes 4-6 showed precise AtMOC1 cleavage without any template degradation. Lanes 7-9 showed non-specific template degradation with higher AtMOC1 protein concentrations. Whereas non-invaded plasmids did not show any precise cleavage and chopping (Lanes 10-14). Non-invaded circular templates with higher protein showed non-specific chopping (Lanes 15-17). Lane 18 is the plasmid treated with EcoRI restriction enzyme, is the size control for the AtMOC1 cleavage reactions. In all the circular reactions, XmnI restriction enzyme added in the cleavage reaction to release a fragment together with AtMOC1 cleavage. Lane M represents the 1-kb plus DNA marker.

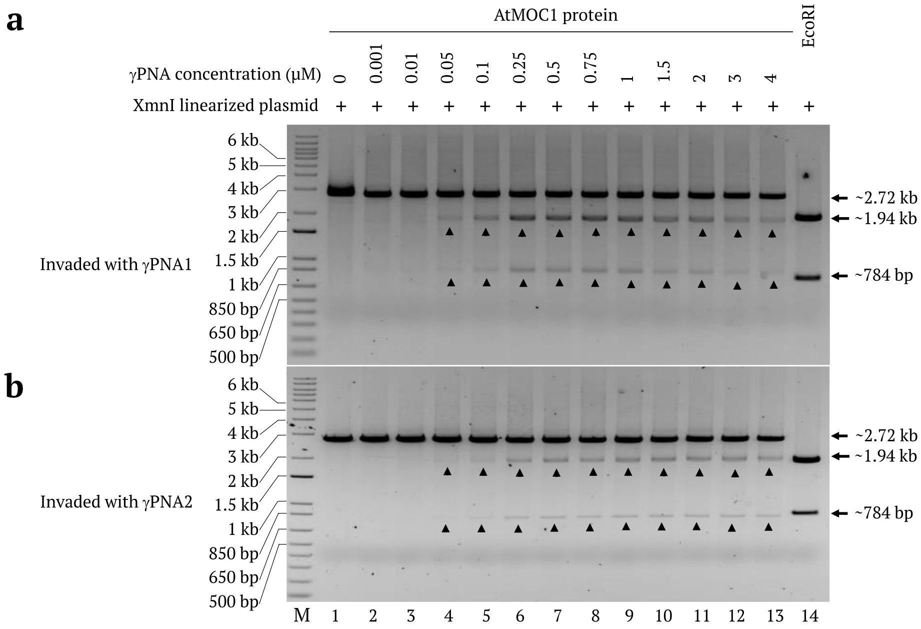

Figure S8. γPNA concentration titration assay linear pUC19-54 via AtMOC1 cleavage. (a) and **(b)** Gel images showing the invasion of different concentrations of γPNA1 and γPNA2 into XmnI linearized plasmids, respectively and treatment with AtMOC1 protein. Lanes 1-13 are the 0 to 4μM concentration of γPNA1 or γPNA2 independent invasion into targets, respectively. Lanes 1-3 did not show any cleavage. Observed template invasion from 50nM concentration (Lane 4) of γPNA1 or γPNA2, evidenced by the AtMOC1 cleavage (Lanes 4-13). Lane 14 is the plasmid treated with EcoRI restriction enzyme, is the size control for the AtMOC1 cleavage reactions. Lane M represents the 1-kb plus DNA marker.

a

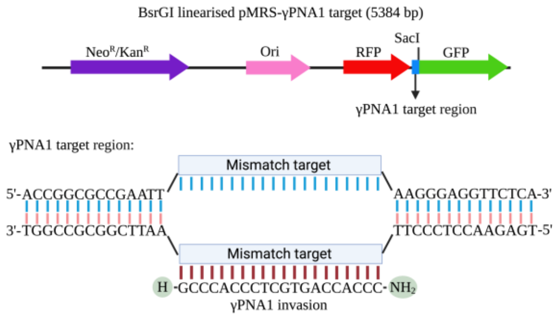

b

No. of mismatches	Target mismatch at 5' end
1	3'CGGGTGGGAGCACTGGTGGC5'
2	3'CGGGTGGGAGCACTGGTGCC5'
3	3'CGGGTGGGAGCACTGGTCCC5'
4	3'CGGGTGGGAGCACTGGACCC5'
5	3'CGGGTGGGAGCACTGCACCC5'
7	3'CGGGTGGGAGCACACCACCC5'
9	3'CGGGTGGGAGCTGACCACCC5'
11	3'CGGGTGGGACGTGACCACCC5'

No. of mismatches	Target mismatch at central region
1	3'CGGGTGGTAGCACTGGTGGG5'
2	3'CGGGTGGTTGCACTGGTGGG5'
3	3'CGGGTGGTTCCACTGGTGGG5'
4	3'CGGGTGGTTCGACTGGTGGG5'

No. of mismatches	No-consecutive target mismatches
2	3'CGGGTGGCACCACTGGTGGG5'
4	3'CGGGTCGCACCTCTGGTGGG5'
6	3'CGGCTCGCACCTCAGGTGGG5'
8	3'CCGCTCGCACCTCAGCTGGG5'

No. of mismatches	Target mismatch at 3' end
1	3'CCGGTGGGAGCACTGGTGGG5'
2	3'CCCGTGGGAGCACTGGTGGG5'
3	3'CCCCTGGGAGCACTGGTGGG5'
4	3'CCCCAGGGAGCACTGGTGGG5'
5	3'CCCCACGGAGCACTGGTGGG5'
7	3'CCCCACCCAGCACTGGTGGG5'
9	3'CCCCACCCTCCACTGGTGGG5'
11	3'CCCCACCCTCGTCTGGTGGG5'

c

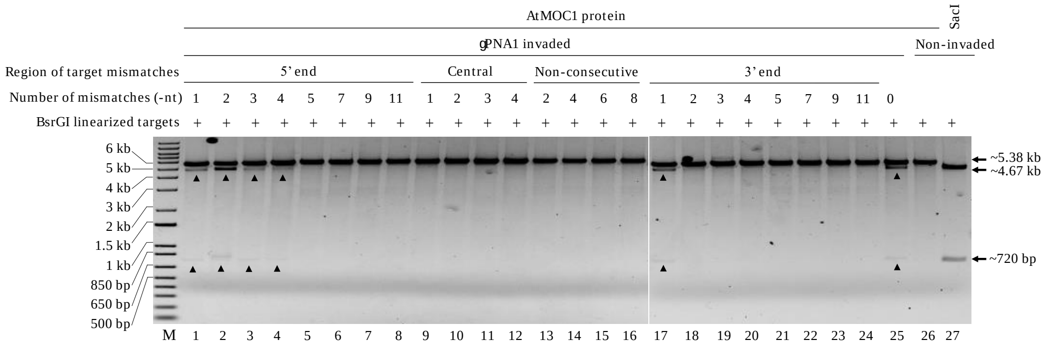

Figure S9. AtMOC1 cleavage of γPNA1 invaded mismatched pMRS targets. (a) Plasmid map showing the BsrGI linearized pMRS plasmid containing the γPNA1 binding region. Sketch representing the γPNA1 invasion into the mismatch target region. **(b)** Table showing the 5' end, 3' end, central, and non-consecutive γPNA1 matched sequences present in the different pMRS targets. **(c)** Gel image showing the AtMOC1 cleavage of γPNA1 invaded different BsrGI linearized pMRS targets, independently. Lanes 1-8 are the AtMOC1 cleavage of γPNA1 invaded 1-nt to 11-nt 5' end mismatched linear targets, respectively. Observed AtMOC1 cleavage in 1-nt to 4-nt 5' end mismatched linear targets (Lanes 1-4). Lanes 9-12 are the AtMOC1 cleavage of γPNA1 invaded 1-nt to 4-nt central region mismatched linear targets, respectively. Lanes 13-16 are the AtMOC1 cleavage of γPNA1 invaded 2-nt to 8-nt non-consecutive mismatched linear targets, respectively. No AtMOC1 cleavage observed in central and non-consecutive mismatched γPNA1 invaded targets (Lanes 9-16). Lanes 17-24 are the AtMOC1 cleavage of γPNA1 invaded 1-nt to 11-nt 3' end mismatched linear targets, respectively. Observed AtMOC1 cleavage in 1-nt 3' end mismatched linear target (Lane 17). Lane 25 is the full γPNA1 complementary target cleavage with AtMOC1. Lane 26 is the non-invaded target cleavage with AtMOC1. Lane 27 is the BsrGI linearized pMRS target restriction cleavage with SacI enzyme as the size control for the AtMOC1 cleavage reactions. Lane M represents the 1-kb plus DNA marker. All samples from this experiment were incubated at the same time. Different gels were used to run all the samples. Later, gel images were assembled together based on the DNA ladder

Supplementary files

1) Protein expression vectors used in this study

A. pCOLD I-AtMOC1

aaggaatggtgtggccgattaatcataaatatgaaaaataattgttgcatcacccgccaatgcgtggctt
aatgcacatcaaattgtgagcggataacaatttgatgtgctagcgcatatccagtgtagtaaggcaagtc
ccttcaagagttatcgttgatacccctcgtagtgcacattcctttaacgcttcaaaatctgtaaagcacg
ccatatcgccgaaaggcacacttaattattaagaggtaatacaccatgaatcacaaagtgcatcatcatc
atcatcatatcgaaggtaggcatatgctggaagttctgttccaggggcccggtggcggtggcagcAGTGC
TCTTCCTACGACGAAAGCCATAGACGCGGCGTTAATGAAGGAGAAATGGTTGGATTCTCTCTCTCTCACC
TCACAAGACGAAGATACGACTCCGGAGAATGCTGAGTCAAGCTGTATCATTGGGATTGACCCTGATTTGT
CTGGTGCGTTGGCTCTTTTGAAATTTGACCACTTGGGTTCTTCTTCTTTTGCTCAGGTTTATGATACACC
TCACATTCCAGTTTTAGTTGGGAAAAGAGTAAGAAAACGTTTGGATGCAAAGTCAATTGTACAATTGATT
CAGAGCTTAGATGTTCCCTCAGGAAGCAGAGTGTATATAGAACAATCGAATCCGTTTCCCAAAGATGGAA
AACAGGGTTGGTATAGTGGAGGATTTGGATATGGATTATGGATAGGAACACTTGTTGCTTCAGGCTTTTG
TGTTATTCCGGTTTCCGCATCTTTATGGAAGAGGCATTTTCAACTTGCTAGTGGAAGTTGCACAAAGGAC
GATAGCAGACGAGTTGCAGCGGAGTTGTTTCCATCGCTTAGTTCGCAACTTAAGAGGAAAAAGGATCATG
GTCGAGCTGAAGCACTGCTCATTGCGGCGTATGGTGAAGCCCTTAAAACAGAGAAATTGTTGATCCAGCC
AAAGGAATTACTCTCTCAAGTTAACTACTTAGAAAACCAGTTAGTGGAGGTTAAAtagaattcaagcttg
tcgacctgcagtctagataggtaatctctgcttaaaagcacagaatctaagatccctgccatttggcggg
gatttttttatttgttttcaggaaataaataatcgatcgcgtaataaaatctattattattttgtgaag
aataaatttgggtgcaatgagaatgcgcaggccctttcgtctcgcgcgtttcggtgatgacggtgaaaac
ctctgacacatgcagctcccggagacggtcacagcttgtctgtaagcggatgccgggagcagacaagccc
gtcagggcgcgtcagcgggtgttggcgggtgtcggggctggcttaactatgcggcatcagagcagattgt
actgagagtgcaccataaaattgtaaacgttaatattttgttaaaattcgcgttaaatttttgttaaatc
agctcattttttaaccaataggccgaaatcggcaaaatcccttataaatcaaaagaatagcccgagatag
ggttgagtgttgttccagtttggaacaagagtccactattaaagaacgtggactccaacgtcaaagggcg
aaaaaccgtctatcagggcgatggcccactacgtgaaccatcacccaaatcaagttttttggggtcgagg
tgccgtaaagcactaaatcggaaccctaaagggagcccccgatttagagcttgacggggaaagccggcga
acgtggcgagaaaggaagggaagaaagcgaaaggagcgggcgctagggcgctggcaagtgtagcggtcac
gctgcgcgtaaccaccacacccgccgcgcttaatgcgccgctacagggcgcgtactatggttgctttgac
gtatgcggtgtgaaataccgcacagatgcgtaaggagaaaataccgcatcaggcgtcaggtggcactttt
cggggaaatgtgcgcggaacccctatttgtttatttttctaaatacattcaaatatgtatccgctcatga
gacaataaccctgataaatgcttcaataatattgaaaaaggaagagtatgagtattcaacatttccgtgt
cgcccttattcccttttttgcggcattttgccttcctgtttttgctcacccagaaacgctggtgaaagta
aaagatgctgaagatcagttgggtgcacgagtgggttacatcgaactggatctcaacagcggtaagatcc
ttgagagttttcgccccgaagaacgttttccaatgatgagcacttttaaagttctgctatgtggcgcggt
attatcccgtattgacgccgggcaagagcaactcggtcgccgcatacactattctcagaatgacttggtt
gagtactcaccagtcacagaaaagcatcttacggatggcatgacagtaagagaattatgcagtgctgcca
taaccatgagtgataacactgcggccaacttacttctgacaacgatcggaggaccgaaggagctaaccgc
ttttttgcacaacatgggggatcatgtaactcgccttgatcgttgggaaccggagctgaatgaagccata
ccaaacgacgagcgtgacaccacgatgcctgtagcaatggcaacaacgttgcgcaaactattaactggcg
aactacttactctagcttcccggcaacaattaatagactggatggaggcggataaagttgcaggaccact
tctgcgctcggcccttccggctggctggtttattgctgataaatctggagccggtgagcgtgggtctcgc
ggtatcattgcagcactggggccagatggtaagccctcccgtatcgtagttatctacacgacggggagtc
aggcaactatggatgaacgaaatagacagatcgctgagataggtgcctcactgattaagcattggtaact
gtcagaccaagtttactcatatatactttagattgatttaaaacttcatttttaatttaaaaggatctag
gtgaagatcctttttgataatctcatgaccaaaatcccttaacgtgagttttcgttccactgagcgtcag
accccgtagaaaagatcaaaggatcttcttgagatcctttttttctgcgcgtaatctgctgcttgcaaac

aaaaaaaccaccgctaccagcggtggtttgtttgccggatcaagagctaccaactctttttccgaaggta
actggcttcagcagagcgcagataccaaatactgttcttctagtgtagccgtagttaggccaccacttca
agaactctgtagcaccgcctacatacctcgctctgctaatcctgttaccagtggctgctgccagtggcga
taagtcgtgtcttaccgggttggactcaagacgatagttaccggataaggcgcagcggtcgggctgaacg
gggggttcgtgcacacagcccagcttggagcgaacgacctacaccgaactgagatacctacagcgtgagc
tatgagaaagcgccacgcttcccgaagggagaaaggcggacaggtatccggtaagcggcagggtcggaac
aggagagcgcacgagggagcttccagggggaaacgcctggtatctttatagtcctgtcgggtttcgccac
ctctgacttgagcgtcgatttttgtgatgctcgtcaggggggcggagcctatggaaaaacgccagcaacg
cggcctttttacggttcctggccttttgctggccttttgctcacatagtcatgccccgcgcccaccggaa
ggagctgactgggttgaaggctctcaagggcatcggtcgagatcccggtgcctaatgagtgagctaactt
acattaattgcgttgcgctcactgcccgctttccagtcgggaaacctgtcgtgccagctgcattaatgaa
tcggccaacgcgcggggagaggcggtttgcgtattgggcgccagggtggtttttcttttcaccagtgaga
cgggcaacagctgattgcccttcaccgcctggccctgagagagttgcagcaagcggtccacgctggtttg
ccccagcaggcgaaaatcctgtttgatggtggttaacggcgggatataacatgagctgtcttcggtatcg
tcgtatcccactaccgagatatccgcaccaacgcgcagcccggactcggtaatggcgcgcattgcgccca
gcgccatctgatcgttggcaaccagcatcgcagtgggaacgatgccctcattcagcatttgcatggtttg
ttgaaaaccggacatggcactccagtcgccttcccgttccgctatcggctgaatttgattgcgagtgaga
tatttatgccagccagccagacgcagacgcgccgagacagaacttaatgggcccgctaacagcgcgattt
gctggtgacccaatgcgaccagatgctccacgcccagtcgcgtaccgtcttcatgggagaaaataatact
gttgatgggtgtctggtcagagacatcaagaaataacgccggaacattagtgcaggcagcttccacagca
atggcatcctggtcatccagcggatagttaatgatcagcccactgacgcgttgcgcgagaagattgtgca
ccgccgctttacaggcttcgacgccgcttcgttctaccatcgacaccaccacgctggcacccagttgatc
ggcgcgagatttaatcgccgcgacaatttgcgacggcgcgtgcagggccagactggaggtggcaacgcca
atcagcaacgactgtttgcccgccagttgttgtgccacgcggttgggaatgtaattcagctccgccatcg
ccgcttccactttttcccgcgttttcgcagaaacgtggctggcctggttcaccacgcgggaaacggtctg
ataagagacaccggcatactctgcgacatcgtataacgttactggtttcacattcaccaccctgaattga
ctctcttccgggcgctatcatgccataccgcgaaaggttttgcgccattcgatggtgtccgggatctcga
cgctctcccttatgcgactcctgcattaggaagcagcccagtagtaggttgaggccgttgagcaccgccg
ccgc

Uppercase and underlined sequence represents AtMOC1 gene sequence

NdeI and EcoRI restriction sites represented in highlighted sequences

2) Target plasmids used in this study

A. pUC19 plasmid used to clone all γPNA target regions between EcoRI and BamHI restriction sites (highlighted) (2,686 bp)

TCGCGCGTTTCGGTGATGACGGTGAAAACCTCTGACACATGCAGCTCCCGGAGACGGTCACAGCTTGTCT
GTAAGCGGATGCCGGGAGCAGACAAGCCCGTCAGGGCGCGTCAGCGGGTGTTGGCGGGTGTCGGGGCTGG
CTTAACTATGCGGCATCAGAGCAGATTGTACTGAGAGTGCACCATATGCGGTGTGAAATACCGCACAGAT
GCGTAAGGAGAAAATACCGCATCAGGCGCCATTCGCCATTCAGGCTGCGCAACTGTTGGGAAGGGCGATC
GGTGCGGGCCTCTTCGCTATTACGCCAGCTGGCGAAAGGGGGATGTGCTGCAAGGCGATTAAGTTGGGTA
ACGCCAGGGTTTTCCCAGTCACGACGTTGTAAAACGACGGCCAGTGAATTCGAGCTCGGTACCCGGGGAT
CCTCTAGAGTCGACCTGCAGGCATGCAAGCTTGGCGTAATCATGGTCATAGCTGTTTCCTGTGTGAAATT
GTTATCCGCTCACAATTCCACACAACATACGAGCCGGAAGCATAAAGTGTAAAGCCTGGGGTGCCTAATG
AGTGAGCTAACTCACATTAATTGCGTTGCGCTCACTGCCCGCTTTCCAGTCGGGAAACCTGTCGTGCCAG
CTGCATTAATGAATCGGCCAACGCGCGGGGAGAGGCGGTTTGCGTATTGGGCGCTCTTCCGCTTCCTCGC
TCACTGACTCGCTGCGCTCGGTCGTTCGGCTGCGGCGAGCGGTATCAGCTCACTCAAAGGCGGTAATACG

GTTATCCACAGAATCAGGGGATAACGCAGGAAAGAACATGTGAGCAAAAGGCCAGCAAAAGGCCAGGAAC
CGTAAAAAGGCCGCGTTGCTGGCGTTTTTCCATAGGCTCCGCCCCCCTGACGAGCATCACAAAAATCGAC
GCTCAAGTCAGAGGTGGCGAAACCCGACAGGACTATAAAGATACCAGGCGTTTCCCCCTGGAAGCTCCCT
CGTGCGCTCTCCTGTTCCGACCCTGCCGCTTACCGGATACCTGTCCGCCTTTCTCCCTTCGGGAAGCGTG
GCGCTTTCTCATAGCTCACGCTGTAGGTATCTCAGTTCGGTGTAGGTCGTTCGCTCCAAGCTGGGCTGTG
TGCACGAACCCCCCGTTCAGCCCGACCGCTGCGCCTTATCCGGTAACTATCGTCTTGAGTCCAACCCGGT
AAGACACGACTTATCGCCACTGGCAGCAGCCACTGGTAACAGGATTAGCAGAGCGAGGTATGTAGGCGGT
GCTACAGAGTTCTTGAAGTGGTGGCCTAACTACGGCTACACTAGAAGAACAGTATTTGGTATCTGCGCTC
TGCTGAAGCCAGTTACCTTCGGAAAAAGAGTTGGTAGCTCTTGATCCGGCAAACAAACCACCGCTGGTAG
CGGTGGTTTTTTTGTTTGCAAGCAGCAGATTACGCGCAGAAAAAAAGGATCTCAAGAAGATCCTTTGATC
TTTTCTACGGGGTCTGACGCTCAGTGGAACGAAAACTCACGTTAAGGGATTTTGGTCATGAGATTATCAA
AAAGGATCTTCACCTAGATCCTTTTAAATTAAAAATGAAGTTTTAAATCAATCTAAAGTATATATGAGTA
AACTTGGTCTGACAGTTACCAATGCTTAATCAGTGAGGCACCTATCTCAGCGATCTGTCTATTTCGTTCA
TCCATAGTTGCCTGACTCCCCGTCGTGTAGATAACTACGATACGGGAGGGCTTACCATCTGGCCCCAGTG
CTGCAATGATACCGCGAGACCCACGCTCACCGGCTCCAGATTTATCAGCAATAAACCAGCCAGCCGGAAG
GGCCGAGCGCAGAAGTGGTCCTGCAACTTTATCCGCCTCCATCCAGTCTATTAATTGTTGCCGGGAAGCT
AGAGTAAGTAGTTCGCCAGTTAATAGTTTGCGCAACGTTGTTGCCATTGCTACAGGCATCGTGGTGTCAC
GCTCGTCGTTTGGTATGGCTTCATTCAGCTCCGGTTCCCAACGATCAAGGCGAGTTACATGATCCCCCAT
GTTGTGCAAAAAAGCGGTTAGCTCCTTCGGTCCTCCGATCGTTGTCAGAAGTAAGTTGGCCGCAGTGTTA
TCACTCATGGTTATGGCAGCACTGCATAATTCTCTTACTGTCATGCCATCCGTAAGATGCTTTTCTGTGA
CTGGTGAGTACTCAACCAAGTCATTCTGAGAATAGTGTATGCGGCGACCGAGTTGCTCTTGCCCGGCGTC
AATACGGGATAATACCGCGCCACATAGCAGAACTTTAAAAGTGCTCATCATTGGAAAACGTTCTTCGGGG
CGAAAACTCTCAAGGATCTTACCGCTGTTGAGATCCAGTTCGATGTAACCCACTCGTGCACCCAACTGAT
CTTCAGCATCTTTTACTTTCACCAGCGTTTCTGGGTGAGCAAAAACAGGAAGGCAAAATGCCGCAAAAAA
GGGAATAAGGGCGACACGGAAATGTTGAATACTCATACTCTTCCTTTTTCAATATTATTGAAGCATTTAT
CAGGGTTATTGTCTCATGAGCGGATACATATTTGAATGTATTTAGAAAAATAAACAAATAGGGGTTCCGC
GCACATTTCCCCGAAAAGTGCCACCTGACGTCTAAGAAACCATTATTATCATGACATTAACCTATAAAAA
TAGGCGTATCACGAGGCCCTTTCGTC

B. Modified pMRS plasmid used to clone all γPNA target regions between EcoRI and BamHI restriction sites (highlighted) (5418 bp)

TAGTTATTAATAGTAATCAATTACGGGGTCATTAGTTCATAGCCCATATATGGAGTTCCGCGTTACATAA
CTTACGGTAAATGGCCCGCCTGGCTGACCGCCCAACGACCCCCGCCCATTGACGTCAATAATGACGTATG
TTCCCATAGTAACGCCAATAGGGACTTTCCATTGACGTCAATGGGTGGAGTATTTACGGTAAACTGCCCA
CTTGGCAGTACATCAAGTGTATCATATGCCAAGTACGCCCCCTATTGACGTCAATGACGGTAAATGGCCC
GCCTGGCATTATGCCCAGTACATGACCTTATGGGACTTTCCTACTTGGCAGTACATCTACGTATTAGTCA
TCGCTATTACCATGGTGATGCGGTTTTGGCAGTACATCAATGGGCGTGGATAGCGGTTTGACTCACGGGG
ATTTCCAAGTCTCCACCCCATTGACGTCAATGGGAGTTTGTTTTGGCACCAAAATCAACGGGACTTTCCA
AAATGTCGTAACAACTCCGCCCCATTGACGCAAATGGGCGGTAGGCGTGTACGGTGGGAGGTCTATATAA
GCAGAGCTGGTTTAGTGAACCGTCAGATCCGCTTGCCACCATGGCCTCCTCCGAGGACGTCATCAAGGAG
TTCATGCGCTTCAAGGTGCGCATGGAGGGCTCCGTGAACGGCCACGAGTTCGAGATCGAGGGCGAGGGCG
AGGGCCGCCCCTACGAGGGCACCCAGACCGCCAAGCTGAAGGTGACCAAGGGCGGCCCCCTGCCCTTCGC
CTGGGACATCCTGTCCCCTCAGTTCCAGTACGGCTCCAAGGCCTACGTGAAGCACCCCGCCGACATCCCC
GACTACTTGAAGCTGTCCTTCCCCGAGGGCTTCAAGTGGGAGCGCGTGATGAACTTCGAGGACGGCGGCG
TGGTGACCGTGACCCAGGACTCCTCCCTGCAGGACGGCGAGTTCATCTACAAGGTGAAGCTGCGCGGCAC
CAACTTCCCCTCCGACGGCCCCGTAATGCAGAAGAAGACCATGGGCTGGGAGGCCTCCACCGAGCGGATG
TACCCCGAGGACGGCGCCCTGAAGGGCGAGATCAAGATGAGGCTGAAGCTGAAGGACGGCGGCCACTACG
ACGCCGAGGTCAAGACCACCTACATGGCCAAGAAGCCCGTGCAGCTGCCCGGCGCCTACAAGACCGACAT
CAAGCTGGACATCACCTCCCACAACGAGGACTACACCATCGTGGAACAGTACGAGCGCGCCGAGGGCCGC
CACTCCACCGGCGCCGAATTCCCGCTAGTGCGATGGCCTCATGGAAGCTTGATATCCAGCCAGGACAATT

TACCGATCCATGAGGCCATCGCACTAGGGGGGATCCAGTGAGCAAGGGCGAGGAGCTGTTCACCGGGGTG
GTGCCCATCCTGGTCGAGCTGGACGGCGACGTAAACGGCCACAAGTTCAGCGTGTCCGGCGAGGGCGAGG
GCGATGCCACCTACGGCAAGCTGACCCTGAAGTTCATCTGCACCACCGGCAAGCTGCCCGTGCCCTGGCC
TACACTGGTTACTACATTAACATATGGAGTCCAATGTTTTAGCCGCTACCCCGACCACATGAAGCAGCAC
GACTTCTTCAAGTCCGCCATGCCCGAAGGCTACGTCCAGGAGCGCACCATCTTCTTCAAGGACGACGGCA
ACTACAAGACCCGCGCCGAGGTGAAGTTCGAGGGCGACACCCTGGTGAACCGCATCGAGCTGAAGGGCAT
CGACTTCAAGGAGGACGGCAACATCCTGGGGCACAAGCTGGAGTACAACTACAACAGCCACAACGTCTAT
ATCATGGCCGACAAGCAGAAGAACGGCATCAAGGTGAACTTCAAGATCCGCCACAACATCGAGGACGGCA
GCGTGCAGCTCGCCGACCACTACCAGCAGAACACCCCCATCGGCGACGGCCCCGTGCTGCTGCCCGACAA
CCACTACCTGAGCACCCAGTCCGCCCTGAGCAAAGACCCCAACGAGAAGCGCGATCACATGGTCCTGCTG
GAGTTCGTGACCGCCGCCGGGATCACTCTCGGCATGGACGAGCTGTACAAGTAAGCGGCCGCGACTCTAG
ATCATAATCAGCCATACCACATTTGTAGAGGTTTTACTTGCTTTAAAAAACCTCCCACACCTCCCCCTGA
ACCTGAAACATAAAATGAATGCAATTGTTGTTGTTAACTTGTTTATTGCAGCTTATAATGGTTACAAATA
AAGCAATAGCATCACAAATTTCACAAATAAAGCATTTTTTTCACTGCATTCTAGTTGTGGTTTGTCCAAA
CTCATCAATGTATCTTAAGGCGTAAATTGTAAGCGTTAATATTTTGTTAAAATTCGCGTTAAATTTTTGT
TAAATCAGCTCATTTTTTAACCAATAGGCCGAAATCGGCAAAATCCCTTATAAATCAAAAGAATAGACCG
AGATAGGGTTGAGTGTTGTTCCAGTTTGGAACAAGAGTCCACTATTAAAGAACGTGGACTCCAACGTCAA
AGGGCGAAAAACCGTCTATCAGGGCGATGGCCCACTACGTGAACCATCACCCTAATCAAGTTTTTTGGGG
TCGAGGTGCCGTAAAGCACTAAATCGGAACCCTAAAGGGAGCCCCCGATTTAGAGCTTGACGGGGAAAGC
CGGCGAACGTGGCGAGAAAGGAAGGGAAGAAAGCGAAAGGAGCGGGCGCTAGGGCGCTGGCAAGTGTAGC
GGTCACGCTGCGCGTAACCACCACACCCGCCGCGCTTAATGCGCCGCTACAGGGCGCGTCAGGTGGCACT
TTTCGGGGAAATGTGCGCGGAACCCCTATTTGTTTATTTTTCTAAATACATTCAAATATGTATCCGCTCA
TGAGACAATAACCCTGATAAATGCTTCAATAATATTGAAAAAGGAAGAGTCCTGAGGCGGAAAGAACCAG
CTGTGGAATGTGTGTCAGTTAGGGTGTGGAAAGTCCCCAGGCTCCCCAGCAGGCAGAAGTATGCAAAGCA
TGCATCTCAATTAGTCAGCAACCAGGTGTGGAAAGTCCCCAGGCTCCCCAGCAGGCAGAAGTATGCAAAG
CATGCATCTCAATTAGTCAGCAACCATAGTCCCGCCCCTAACTCCGCCCATCCCGCCCCTAACTCCGCCC
AGTTCCGCCCATTCTCCGCCCCATGGCTGACTAATTTTTTTTATTTATGCAGAGGCCGAGGCCGCCTCGG
CCTCTGAGCTATTCCAGAAGTAGTGAGGAGGCTTTTTTGGAGGCCTAGGCTTTTGCAAAGATCGATCAAG
AGACAGGATGAGGATCGTTTCGCATGATTGAACAAGATGGATTGCACGCAGGTTCTCCGGCCGCTTGGGT
GGAGAGGCTATTCGGCTATGACTGGGCACAACAGACAATCGGCTGCTCTGATGCCGCCGTGTTCCGGCTG
TCAGCGCAGGGGCGCCCGGTTCTTTTTGTCAAGACCGACCTGTCCGGTGCCCTGAATGAACTGCAAGACG
AGGCAGCGCGGCTATCGTGGCTGGCCACGACGGGCGTTCCTTGCGCAGCTGTGCTCGACGTTGTCACTGA
AGCGGGAAGGGACTGGCTGCTATTGGGCGAAGTGCCGGGGCAGGATCTCCTGTCATCTCACCTTGCTCCT
GCCGAGAAAGTATCCATCATGGCTGATGCAATGCGGCGGCTGCATACGCTTGATCCGGCTACCTGCCCAT
TCGACCACCAAGCGAAACATCGCATCGAGCGAGCACGTACTCGGATGGAAGCCGGTCTTGTCGATCAGGA
TGATCTGGACGAAGAGCATCAGGGGCTCGCGCCAGCCGAACTGTTCGCCAGGCTCAAGGCGAGCATGCCC
GACGGCGAGGATCTCGTCGTGACCCATGGCGATGCCTGCTTGCCGAATATCATGGTGGAAAATGGCCGCT
TTTCTGGATTCATCGACTGTGGCCGGCTGGGTGTGGCGGACCGCTATCAGGACATAGCGTTGGCTACCCG
TGATATTGCTGAAGAGCTTGGCGGCGAATGGGCTGACCGCTTCCTCGTGCTTTACGGTATCGCCGCTCCC
GATTCGCAGCGCATCGCCTTCTATCGCCTTCTTGACGAGTTCTTCTGAGCGGGACTCTGGGGTTCGAAAT
GACCGACCAAGCGACGCCCAACCTGCCATCACGAGATTTCGATTCCACCGCCGCCTTCTATGAAAGGTTG
GGCTTCGGAATCGTTTTCCGGGACGCCGGCTGGATGATCCTCCAGCGCGGGGATCTCATGCTGGAGTTCT
TCGCCCACCCTAGGGGGAGGCTAACTGAAACACGGAAGGAGACAATACCGGAAGGAACCCGCGCTATGAC
GGCAATAAAAAGACAGAATAAAACGCACGGTGTTGGGTCGTTTGTTCATAAACGCGGGGTTCGGTCCCAG
GGCTGGCACTCTGTCGATACCCCACCGAGACCCCATTGGGGCCAATACGCCCGCGTTTCTTCCTTTTCCC
CACCCCACCCCCCAAGTTCGGGTGAAGGCCCAGGGCTCGCAGCCAACGTCGGGGCGGCAGGCCCTGCCAT
AGCCTCAGGTTACTCATATATACTTTAGATTGATTTAAAACTTCATTTTTAATTTAAAAGGATCTAGGTG
AAGATCCTTTTTGATAATCTCATGACCAAAATCCCTTAACGTGAGTTTTCGTTCCACTGAGCGTCAGACC
CCGTAGAAAAGATCAAAGGATCTTCTTGAGATCCTTTTTTTCTGCGCGTAATCTGCTGCTTGCAAACAAA
AAAACCACCGCTACCAGCGGTGGTTTGTTTGCCGGATCAAGAGCTACCAACTCTTTTTCCGAAGGTAACT
GGCTTCAGCAGAGCGCAGATACCAAATACTGTCCTTCTAGTGTAGCCGTAGTTAGGCCACCACTTCAAGA
ACTCTGTAGCACCGCCTACATACCTCGCTCTGCTAATCCTGTTACCAGTGGCTGCTGCCAGTGGCGATAA

GTCGTGTCTTACCGGGTTGGACTCAAGACGATAGTTACCGGATAAGGCGCAGCGGTCGGGCTGAACGGGG
GGTTCGTGCACACAGCCCAGCTTGGAGCGAACGACCTACACCGAACTGAGATACCTACAGCGTGAGCTAT
GAGAAAGCGCCACGCTTCCCGAAGGGAGAAAGGCGGACAGGTATCCGGTAAGCGGCAGGGTCGGAACAGG
AGAGCGCACGAGGGAGCTTCCAGGGGGAAACGCCTGGTATCTTTATAGTCCTGTCGGGTTTCGCCACCTC
TGACTTGAGCGTCGATTTTTGTGATGCTCGTCAGGGGGGCGGAGCCTATGGAAAAACGCCAGCAACGCGG
CCTTTTTACGGTTCCTGGCCTTTTGCTGGCCTTTTGCTCACATGTTCTTTCCTGCGTTATCCCCTGATTC
TGTGGATAACCGTATTACCGCCATGCAT

C. pUC19-Multiplexing target-151 plasmid (γPNA binding sites are highlighted) (3960 bp)

TCGCGCGTTTCGGTGATGACGGTGAAAACCTCTGACACATGCAGCTCCCGGAGACGGTCACAGCTTGTCT
GTAAGCGGATGCCGGGAGCAGACAAGCCCGTCAGGGCGCGTCAGCGGGTGTTGGCGGGTGTCGGGGCTGG
CTTAACTATGCGGCATCAGAGCAGATTGTACTGAGAGTGCACCATACACAGGGTGGTCACGAGGGTGGGC
TGACTGTACAACATCCGCATTGAGAACCTCCCTTGAGTATGCGGTGTGAAATACCGCACAGATGCGTAAG
GAGAAAATACCGCATCAGGCGCCATTCGCCATTCAGGCTGCGCAACTGTTGGGAAGGGCGATCGGTGCGG
GCCTCTTCGCTATTACGCCAGCTGGCGAAAGGGGGATGTGCTGCAAGGCGATTAAGTTGGGTAACGCCAG
GGTTTTCCCAGTCACGACGTTGTAAAACGACGGCCAGTGAATTCGAACGCGCAATCAAAACTAAATACGG
TAGCGATACCGAGATCAAGCTCAAATCCAAATCTGGGATTATGCATGACTCCAAATATTTGGAATCATGG
GAGCGGGGCAGTGCGGATATCCGTTTCGCAGAGTTCGCCGGCGAGAATCGAGCTCACAACAAGCAGTTTC
CGGCTGCGACTGTGAATATGGGAAGGCAGCCAGATGGCCAGGGAGGGATGACTCGCGATCGCCATGTAAG
CGTTGACTACCTATTGCAAAACCTACCCAACTCCCCTTGGACGCAAGCCTTGAAAGAGGGAAAGTTGTGG
GATCGAGTTCAGGTCCTTGCTCGCGACGGAAACCGTTACATGTCACCTTCAAGACTGGAATATTCCGACC
CCGAACACTTTACCCAACTGATGGATCAAGTTGGTCTGCCCGTGTCGATGGGTCGGCAAAGTCATGCGAA
TAGTGTCAAGTTTGAGCAGTTTGACAGACAGGCAGCGGTTATTGTTGCGGATGGCCCGAACTTACGTGAG
GTTCCAGATTTGTCCCCGGAAAAGTTGCAACAACTGTCTCAAAAAGATGTCCTGATAGCGGATCGCAATG
AAAAGGGGCAAAGAACCGGCACTTACACTAATGTTGTGGAATATGAGCGCCTGATGATGAAATTACCGAG
CGACGCAGCGCAGCTTCTCGCTGAACCGTCCGATAGATATTCACGTGCTTTTGTCCGGCCGGAGCCAGCA
TTGCCCCCCATCAGTGACAGCCGGCGGACTTATGAAAGCCGACCGCGCGGCCCAACCGTAAACAGTCTGA
AAAGGCCGGCGGCCACGAAAAAGGCCGGCCAGGCAAAAAAGAAAAAGTAGGCGGCCGCACTCGAGGCCCG
AAAGGAAGCTGAGTTGGCTGCTGCCACCGCTGAGCAATAACTAGCATAACCCCTTGGGGCCTCTAAACGG
GTCTTGAGGGGTTTTTTGCTGAAAGGAGGAACTATATCCGGATATCCCGCAAGAGGCCCGGCAGTACCGG
CATAACCAAGCCTATGCCTACAGCATCCAGGGTGACGGTGCCGAGGATGACGATGAGCGCATTGTTAGAT
TTCATACACGGTGCCTGACTGCGTTAGCAATTTAACTGTGATAAACTACCGCATTAAAGCTTATCGATGA
TAAGCTGTCAAACATGAGAATTCACCTGAGTCCGAGCAGAAGAAGAGGAGAGCTCACCGGCTCCCATCAC
ATCAACCGGAGGATCCTCTAGAGTCGACCTGCAGGCATGCAAGCTTGGCGTAATCATGGTCATAGCTGTT
TCCTGTGTGAAATTGTTATCCGCTCACAATTCCACACAACATACGAGCCGGAAGCATAAAGTGTAAAGCC
TGGGGTGCCTAATGAGTGAGCTAACTCACATTAATTGCGTTGCGCTCACTGCCCGCTTTCCAGTCGGGAA
ACCTGTCGTGCCAGCTGCATTAATGAATCGGCCAACGCGCGGGGAGAGGCGGTTTGCGTATTGGGCGCTC
TTCCGCTTCCTCGCTCACTGACTCGCTGCGCTCGGTCGTTCGGCTGCGGCGAGCGGTATCAGCTCACTCA
AAGGCGGTAATACGGTTATCCACAGAATCAGGGGATAACGCAGGAAAGAACATGTGAGCAAAAGGCCAGC
AAAAGGCCAGGAACCGTAAAAAGGCCGCGTTGCTGGCGTTTTTCCATAGGCTCCGCCCCCCTGACGAGCA
TCACAAAAATCGACGCTCAAGTCAGAGGTGGCGAAACCCGACAGGACTATAAAGATACCAGGCGTTTCCC
CCTGGAAGCTCCCTCGTGCGCTCTCCTGTTCCGACCCTGCCGCTTACCGGATACCTGTCCGCCTTTCTCC
CTTCGGGAAGCGTGGCGCTTTCTCATAGCTCACGCTGTAGGTATCTCAGTTCGGTGTAGGTCGTTCGCTC
CAAGCTGGGCTGTGTGCACGAACCCCCCGTTCAGCCCGACCGCTGCGCCTTATCCGGTAACTATCGTCTT
GAGTCCAACCCGGTAAGACACGACTTATCGCCACTGGCAGCAGCCACTGGTAACAGGATTAGCAGAGCGA
GGTATGTAGGCGGTGCTACAGAGTTCTTGAAGTGGTGGCCTAACTACGGCTACACTAGAAGAACAGTATT
TGGTATCTGCGCTCTGCTGAAGCCAGTTACCTTCGGAAAAAGAGTTGGTAGCTCTTGATCCGGCAAACAA
ACCACCGCTGGTAGCGGTGGTTTTTTTGTTTGCAAGCAGCAGATTACGCGCAGAAAAAAAGGATCTCAAG
AAGATCCTTTGATCTTTTCTACGGGGTCTGACGCTCAGTGGAACGAAAACTCACGTTAAGGATTTTGGT
CATGAGATTATCAAAAAGGATCTTCACCTAGATCCTTTTAAATTAAAAATGAAGTTTTAAATCAATCTAA

AGTATATATGAGTAAACTTGGTCTGACAGTTACCAATGCTTAATCAGTGAGGCACCTATCTCAGCGATCT
GTCTATTTCGTTCATCCATAGTTGCCTGACTCCCCGTCGTGTAGATAACTACGATACGGGAGGGCTTACC
ATCTGGCCCCAGTGCTGCAATGATACCGCGAGACCCACGCTCACCGGCTCCAGATTTATCAGCAATAAAC
CAGCCAGCCGGAAGGGCCGAGCGCAGAAGTGGTCCTGCAACTTTATCCGCCTCCATCCAGTCTATTAATT
GTTGCCGGGAAGCTAGAGTAAGTAGTTCGCCAGTTAATAGTTTGCGCAACGTTGTTGCCATTGCTACAGG
CATCGTGGTGTCACGCTCGTCGTTTGGTATGGCTTCATTCAGCTCCGGTTCCCAACGATCAAGGCGAGTT
ACATGATCCCCCATGTTGTGCAAAAAAGCGGTTAGCTCCTTCGGTCCTCCGATCGTTGTCAGAAGTAAGT
TGGCCGCAGTGTTATCACTCATGGTTATGGCAGCACTGCATAATTCTCTTACTGTCATGCCATCCGTAAG
ATGCTTTTCTGTGACTGGTGAGTACTCAACCAAGTCATTCTGAGAATAGTGTATGCGGCGACCGAGTTGC
TCTTGCCCGGCGTCAATACGGGATAATACCGCGCCACATAGCAGAACTTTAAAAGTGCTCATCATTGGAA
AACGTTCTTCGGGGCGAAAACTCTCAAGGATCTTACCGCTGTTGAGATCCAGTTCGATGTAACCCACTCG
TGCACCCAACTGATCTTCAGCATCTTTTACTTTCACCAGCGTTTCTGGGTGAGCAAAAACAGGAAGGCAA
AATGCCGCAAAAAAGGGAATAAGGGCGACACGGAAATGTTGAATACTCATACTCTTCCTTTTTCAATATT
ATTGAAGCATTTATCAGGGTTATTGTCTCATGAGCGGATACATATTTGAATGTATTTAGAAAAATAAACA
AATAGGGGTTCCGCGCACATTTCCCCGAAAAGTGCCACCTGACGTCTAAGAAACCATTATTATCATGACA
TTAACCTATAAAAATAGGCGTATCACGAGGCCCTTTCGTC

Supplementary tables

Table S1. Primers used in the current study

Primer name	Primer sequence (5’----3’)	Employed for
pCOLD I-Forward	ACGCCATATCGCCGAAAGG	Sanger sequencing of pCOLD I-AtMOC1 expression clones
pCOLD I-Reverse	GGCAGGGATCTTAGATTCTG	
pUC19-1447 Forward	GGGCTGGCTTAACTATGCGG	All the pUC19 target plasmids confirmation by Sanger sequencing, sanger sequencing for cleavage site determination, PCR amplification of pUC19 targets for producing the amplicons for mobility shift assays
pUC19-1448 Reverse	CGAGGAAGCGGAAGAGCG	
pMRS-1442 Forward	GGCCCCGTAATGCAGAAGAAG	pMRS mismatch target plasmids confirmation by Sanger sequencing
pMRS-1444 Reverse	CTCGGCGCGGGTCTTGTAG	

Table S2. AtMOC1 protein sequence

Protein name	Sequence
AtMOC1	SALPTTKAIDAALMKEKWLDSLSLTSQDEDTTPENAESSCIIGIDPDLSGALALLKFD<br>HLGSSSFAQVYDTPHIPVLVGKRVRKRLDAKSIVQLIQSLDVPSGSRVYIEQSNPFPK<br>DGKQGWYSGGFGYGLWIGTLVASGFCVIPVSASLWKRHFQLASGSCTKDDSRRVAAEL<br>FPSLSSQLKRKKDHGRAEALLIAAYGEALKTEKLLIQPKELLSQVNYLENQLVEVK

Table S3. Different PNA sequences tested in the current study

Name	Sequence	PNA modifications	Tm values
γPNA1	H-KKK-GCCCACCCTCGTGACCACCC-KKK-propargylglycine-NH_2	Gamma-alanine at all bases	85.0°C
γPNA2	H-KKK-TCCGCATTGAGAACCTCCCT-KKK-propargylglycine-NH_2	Gamma-alanine at all bases	81.1°C
γPNA3	H-KKK-TCTTCTTCTGCTCGGACTCA-KKK-propargylglycine-NH_2	Gamma-alanine at all bases	75.8°C
γPNA4	H-KKK-GGCTCCCATCACATCAACC-KKK-propargylglycine-NH_2	Gamma-alanine at all bases	78.8°C
γtcPNA1	Cy5-KKK-JTJJJTJJTJTJ-OOO-CT*CT*CC*TC*CC*TC*TT*CG*CC*-KKK-NH_2	X* indicates bases with gamma-alanine	70.9°C
γtcPNA2	Cy5-KKK-JTJJTTJTTJJ-OOO-CC*TT*CT*TC*CT*CG*CG*TC*CT*-KKK-NH_2	X* indicates bases with gamma-alanine	72.5°C
γPNA1-16-nt	H-KKK-GCCCACCCTCGTGACC-KKK-propargylglycine-NH_2	Gamma-alanine at all bases	78.5°C
γPNA1-14-nt	H-KKK-GCCCACCCTCGTGA-KKK-propargylglycine-NH_2	Gamma-alanine at all bases	75.8°C
γPNA1-10-nt	H-KKK-GCCCACCCTC-KKK-propargylglycine-NH_2	Gamma-alanine at all bases	60.0°C
γPNA3-16-nt	H-KKK-TCCGCATTGAGAACCT-KKK-propargylglycine-NH_2	Gamma-alanine at all bases	75.7°C
γPNA3-14-nt	H-KKK-TCCGCATTGAGAAC-KKK-propargylglycine-NH_2	Gamma-alanine at all bases	71.8°C
γPNA3-10-nt	H-KKK-TCCGCATTGA-KKK-propargylglycine-NH_2	Gamma-alanine at all bases	59.6°C

H, free amine at the N terminus.
NH_2, amide at the C terminus.
J, pseudoisocytosine.
K, lysine.
O, 8-Amino-3,6-dioxaoctanoic acid linkers.

Table S4. Top and bottom oligos used to clone the target sequences in pUC19 or pMRS vectors

Oligo name	Oligo sequence (5'---3')	Targets used in figures and supplementary figures
Target 54_20-nt-γPNA1/2_top	AATTCACAGGGTGGTCACGAGGGTGGGCTGAGAGC TCACATCCGCATTGAGAACCTCCCTTGAG	Figures 1c,d; 2c,d; 3a,b; 4c,d; 5c,d; 6a,b; 7a,b; 8a-d; S2c,d; S5a,b; S6a,b; S7a,b
Target 54_ 20-nt-γPNA1/2_bottom	GATCCTCAAGGGAGGTTCTCAATGCGGATGTGAGC TCTCAGCCCACCCTCGTGACCACCCTGTG	Figures 1c,d; 2c,d; 3a,b; 4c,d; 5c,d; 6a,b; 7a,b; 8a-d; S2c,d; S5a,b; S6a,b; S7a,b
Target 49_ γPNA3/4_top	AATTCACCTGAGTCCGAGCAGAAGAAGAGGAGAGC TCACCGGCTCCCATCACATCAACCGGAG	Figures 1c,d; S4c
Target 49_ γPNA3/4_bottom	GATCCTCCGGTTGATGTGATGGGAGCCGGTGAGCT CTCCTCTTCTTCTGCTCGGACTCAGGTG	Figures 1c,d; S4c
Target 47_ γPNA1/2_top	AATTCACCGGGTGGTCACGAGGGTGGGCGGAGAGC TCACCTCCGCATTGAGAACCTCCCTGGAG	Figure 2c,d
Target 47_ γPNA1/2_bottom	GATCCTCCAGGGAGGTTCTCAATGCGGAGGTGAGC TCTCCGCCCACCCTCGTGACCACCCGGTG	Figure 2c,d
Target 51_ γPNA1/2_top	AATTCAGGCCGGGTGGTCACGAGGGTGGGCGGCCA GAGCTCAGGCCTCCGCATTGAGAACCTCCCTGGCC AG	Figure 2c,d
Target 51_ γPNA1/2_bottom	GATCCTGGCCAGGGAGGTTCTCAATGCGGAGGCCT GAGCTCTGGCCGCCCACCCTCGTGACCACCCGGCC TG	Figure 2c,d
Target 52_ γPNA1/2_top	AATTCAATGTCCTAGCAAGGGTGGTCACGAGGGTG GGCTTGCTAGGACATAGAGCTCAATGTCCTAGCAA TCCGCATTGAGAACCTCCCTTTGCTAGGACATAG	Figure 2c,d
Target 52_ γPNA1/2_bottom	GATCCTATGTCCTAGCAAAGGGAGGTTCTCAATGC GGATTGCTAGGACATTGAGCTCTATGTCCTAGCAA GCCCACCCTCGTGACCACCCTTGCTAGGACATTG	Figure 2c,d
Target 55_ γPNA1/2_top	AATTCACGGGGTGGTCACGAGGGTGGGCCGAGAGC TCACGTCCGCATTGAGAACCTCCCTCGAG	Figure 2c,d

Target 55_ γPNA1/2_bottom	GATCCTCGAGGGAGGTTCTCAATGCGGACGTGAGC TCTCGGCCCACCCTCGTGACCACCCCGTG	Figure 2c,d
Target 56_ γPNA1/2_top	AATTCACTGGGTGGTCACGAGGGTGGGCAGAGAGC TCACTTCCGCATTGAGAACCTCCCTAGAG	Figure 2c,d
Target 56_ γPNA1/3_bottom	GATCCTCTAGGGAGGTTCTCAATGCGGAAGTGAGC TCTCTGCCCACCCTCGTGACCACCCAGTG	Figure 2c,d
Target 57_ γPNA1/2_top	AATTCAACGGGTGGTCACGAGGGTGGGCGTAGAGC TCAACTCCGCATTGAGAACCTCCCTGTAG	Figure 2c,d
Target 57_ γPNA1/2_bottom	GATCCTACAGGGAGGTTCTCAATGCGGAGTTGAGC TCTACGCCCACCCTCGTGACCACCCGTTG	Figure 2c,d
Target 58_ γPNA1/2_top	AATTCAGCGGGTGGTCACGAGGGTGGGCGCAGAGC TCAGCTCCGCATTGAGAACCTCCCTGCAG	Figures 2c,d; S2c,d
Target 58_ γPNA1/2_bottom	GATCCTGCAGGGAGGTTCTCAATGCGGAGCTGAGC TCTGCGCCCACCCTCGTGACCACCCGCTG	Figures 2c,d; S2c,d
Target 121_ γPNA1/2_top	AATTCAGCGGGTGGTCACGAGGGTGGGCACAGAGC TCAGCTCCGCATTGAGAACCTCCCTACAG	Figure S2c,d
Target 121_ γPNA1/2_bottom	GATCCTGTAGGGAGGTTCTCAATGCGGAGCTGAGC TCTGTGCCCACCCTCGTGACCACCCGCTG	Figure S2c,d
Target 122_ γPNA1/2_top	AATTCAGAGGGTGGTCACGAGGGTGGGCACAGAGC TCAGATCCGCATTGAGAACCTCCCTACAG	Figure S2c,d
Target 122_ γPNA1/2_bottom	GATCCTGTAGGGAGGTTCTCAATGCGGATCTGAGC TCTGTGCCCACCCTCGTGACCACCCTCTG	Figure S2c,d
Target 123_ γPNA1/2_top	AATTCCGCGGGTGGTCACGAGGGTGGGCGCGGAGC TCCGCTCCGCATTGAGAACCTCCCTGCGG	Figure S2c,d
Target 123_ γPNA1/2_bottom	GATCCCGCAGGGAGGTTCTCAATGCGGAGCGGAGC TCCGCGCCCACCCTCGTGACCACCCGCGG	Figure S2c,d
Target 124_ γPNA1/2_top	AATTCGGCGGGTGGTCACGAGGGTGGGCGCCGAGC TCGGCTCCGCATTGAGAACCTCCCTGCCG	Figure S2c,d
Target 124_ γPNA1/2_bottom	GATCCGGCAGGGAGGTTCTCAATGCGGAGCCGAGC TCGGCGCCCACCCTCGTGACCACCCGCCG	Figure S2c,d
Target 125_ γPNA1/2_top	AATTCAGCGGGTGGTCACGAGGGTGGGCGCTGAGC TCAGCTCCGCATTGAGAACCTCCCTGCTG	Figure S2c,d
Target 125_ γPNA1/2_bottom	GATCCAGCAGGGAGGTTCTCAATGCGGAGCTGAGC TCAGCGCCCACCCTCGTGACCACCCGCTG	Figure S2c,d
Target 126_ γPNA1/2_top	AATTCTGCGGGTGGTCACGAGGGTGGGCGCAGAGC TCTGCTCCGCATTGAGAACCTCCCTGCAG	Figure S2c,d
Target 126_ γPNA1/2_bottom	GATCCTGCAGGGAGGTTCTCAATGCGGAGCAGAGC TCTGCGCCCACCCTCGTGACCACCCGCAG	Figure S2c,d
Target 144_ γPNA1/2_top	AATTCACATGGGTGGTCACGAGGGTGGGCTTGAGA GCTCACATTCCGCATTGAGAACCTCCCTTTGAG	Figure S3b
Target 144_ γPNA1/2_bottom	GATCCTCAAAGGGAGGTTCTCAATGCGGAATGTGA GCTCTCAAGCCCACCCTCGTGACCACCCATGTG	Figure S3b
Target 145_ γPNA1/2_top	AATTCACCTGGGTGGTCACGAGGGTGGGCTGGAGA GCTCACCTTCCGCATTGAGAACCTCCCTTGGAG	Figure S3b

Target 145_ γPNA1/2_bottom	GATCCTCCAAGGGAGGTTCTCAATGCGGAAGGTGA GCTCTCCAGCCCACCCTCGTGACCACCCAGGTG	Figure S3b
Target 146_ γPNA1/2_top	AATTCACGTGGGTGGTCACGAGGGTGGGCTCGAGA GCTCACGTTCCGCATTGAGAACCTCCCTTCGAG	Figure S3b
Target 146_ γPNA1/2_bottom	GATCCTCGAAGGGAGGTTCTCAATGCGGAACGTGA GCTCTCGAGCCCACCCTCGTGACCACCCACGTG	Figure S3b
Target 147_ γPNA1/2_top	AATTCACTTGGGTGGTCACGAGGGTGGGCTAGAGA GCTCACTTTCCGCATTGAGAACCTCCCTTAGAG	Figure S3b
Target 147_ γPNA1/2_bottom	GATCCTCTAAGGGAGGTTCTCAATGCGGAAAGTGA GCTCTCTAGCCCACCCTCGTGACCACCCAAGTG	Figure S3b
Target 148_ γPNA1/2_top	AATTCAACTGGGTGGTCACGAGGGTGGGCTGTAGA GCTCAACTTCCGCATTGAGAACCTCCCTTGTAG	Figure S3b
Target 148_ γPNA1/2_bottom	GATCCTACAAGGGAGGTTCTCAATGCGGAAGTTGA GCTCTACAGCCCACCCTCGTGACCACCCAGTTG	Figure S3b
Target 149_ γPNA1/2_top	AATTCAGCTGGGTGGTCACGAGGGTGGGCTGCAGA GCTCAGCTTCCGCATTGAGAACCTCCCTTGCAG	Figure S3b
Target 149_ γPNA1/2_bottom	GATCCTGCAAGGGAGGTTCTCAATGCGGAAGCTGA GCTCTGCAGCCCACCCTCGTGACCACCCAGCTG	Figure S3b
Target 50_ γtcPNA1/2_top	AATTCACCGGCGAAGAGGGAGGAGAGGGAGAGCTC ACCCCTTCTTCCTCGCGTCCTGGAG	Figure 4c
Target 50_ γtcPNA1/2_bottom	GATCCTCCAGGACGCGAGGAAGAAGGGGTGAGCTC TCCCTCTCCTCCCTCTTCGCCGGTG	Figure 4c
Target 141_10-nt- γPNA1/2_top	AATTCACAGAGGGTGGGCTGAGAGCTCACATCCGC ATTGATGAG	Figure 5c,d
Target 141_10-nt γPNA1/2_bottom	GATCCTCATCAATGCGGATGTGAGCTCTCAGCCCA CCCTCTGTG	Figure 5c,d
Target 142_14-nt- γPNA1/2_top	AATTCACATCACGAGGGTGGGCTGAGAGCTCACAT CCGCATTGAGAACTGAG	Figure 5c,d
Target 142_14-nt- γPNA1/2_bottom	GATCCTCAGTTCTCAATGCGGATGTGAGCTCTCAG CCCACCCTCGTGATGTG	Figure 5c,d
Target 143_16-nt- γPNA1/2_top	AATTCACAGGTCACGAGGGTGGGCTGAGAGCTCAC ATCCGCATTGAGAACCTTGAG	Figure 5c,d
Target 143_16-nt- γPNA1/2_bottom	GATCCTCAAGGTTCTCAATGCGGATGTGAGCTCTC AGCCCACCCTCGTGACCTGTG	Figure 5c,d
Multiplex target 151_ γPNA1_top with NdeI	TACACAGGGTGGTCACGAGGGTGGGCTGACTGTAC AACATCCGCATTGAGAACCTCCCTTGAG	Figure 9b,c
Multiplex target 151_ γPNA1_bottom with NdeI	TACTCAAGGGAGGTTCTCAATGCGGATGTTGTACA GTCAGCCCACCCTCGTGACCACCCTGTG	Figure 9b,c
γPNA1/2_both_1-nt mismatch 5' end top	AATTGCCCACCCTCGTGACCACCGATGGGAGGTTC TCAATGCGGAGAGCTC	Figure S8c

γPNA1/2_both_1-nt mismatch 5' end bottom	GATCGAGCTCTCCGCATTGAGAACCTCCCATCGGT GGTCACGAGGGTGGGC	Figure S8c
γPNA1/2_both_2-nt mismatch 5' end top	AATTGCCCACCCTCGTGACCACGGATCGGAGGTTC TCAATGCGGAGAGCTC	Figure S8c
γPNA1/2_both_2-nt mismatch 5' end bottom	GATCGAGCTCTCCGCATTGAGAACCTCCGATCCGT GGTCACGAGGGTGGGC	Figure S8c
γPNA1/2_both_3-nt mismatch 5' end top	AATTGCCCACCCTCGTGACCAGGGATCCGAGGTTC TCAATGCGGAGAGCTC	Figure S8c
γPNA1/2_both_3-nt mismatch 5' end bottom	GATCGAGCTCTCCGCATTGAGAACCTCGGATCCCT GGTCACGAGGGTGGGC	Figure S8c
γPNA1/2_both_4-nt mismatch 5' end top	AATTGCCCACCCTCGTGACCTGGGATCCCAGGTTC TCAATGCGGAGAGCTC	Figure S8c
γPNA1/2_both_4-nt mismatch 5' end bottom	GATCGAGCTCTCCGCATTGAGAACCTGGGATCCCA GGTCACGAGGGTGGGC	Figure S8c
γPNA1/2_both_1-nt mismatch 3' end top	AATTGGCCACCCTCGTGACCACCCAAGGGAGGTTC TCAATGCGGTGAGCTC	Figure S8c
γPNA1/2_both_1-nt mismatch 3' end bottom	GATCGAGCTCACCGCATTGAGAACCTCCCTTGGGT GGTCACGAGGGTGGCC	Figure S8c
γPNA1/2_both_2-nt mismatch 3' end top	AATTGGGCACCCTCGTGACCACCCAAGGGAGGTTC TCAATGCGCTGAGCTC	Figure S8c
γPNA1/2_both_2-nt mismatch 3' end bottom	GATCGAGCTCAGCGCATTGAGAACCTCCCTTGGGT GGTCACGAGGGTGCCC	Figure S8c
γPNA1/2_both_3-nt mismatch 3' end top	AATTGGGGACCCTCGTGACCACCCAAGGGAGGTTC TCAATGCCCTGAGCTC	Figure S8c
γPNA1/2_both_3-nt mismatch 3' end bottom	GATCGAGCTCAGGGCATTGAGAACCTCCCTTGGGT GGTCACGAGGGTCCCC	Figure S8c
γPNA1/2_both_4-nt mismatch 3' end top	AATTGGGGTCCCTCGTGACCACCCAAGGGAGGTTC TCAATGGCCTGAGCTC	Figure S8c
γPNA1/2_both_4-nt mismatch 3' end bottom	GATCGAGCTCAGGCCATTGAGAACCTCCCTTGGGT GGTCACGAGGGACCCC	Figure S8c
γPNA1/2_both_1-nt mismatch top-central	AATTGCCCACCATCGTGACCACCCAAGGGAGGTTA TCAATGCGGAGAGCTC	Figure S8c
γPNA1/2_both_1-nt mismatch bottom-central	GATCGAGCTCTCCGCATTGATAACCTCCCTTGGGT GGTCACGATGGTGGGC	Figure S8c
γPNA1/2_both_2-nt mismatch top-central	AATTGCCCACCAACGTGACCACCCAAGGGAGGTTA ACAATGCGGAGAGCTC	Figure S8c

γPNA1/2_both_2-nt mismatch bottom-central	GATCGAGCTCTCCGCATTGTTAACCTCCCTTGGGTGGTCACGTTGGTGGGC	Figure S8c
γPNA1/2_both_3-nt mismatch top-central	AATTGCCCACCAAGGTGACCACCCAAGGGAGGTTAAGAATGCGGAGAGCTC	Figure S8c
γPNA1/2_both_3-nt mismatch bottom-central	GATCGAGCTCTCCGCATTCTTAACCTCCCTTGGGTGGTCACCTTGGTGGGC	Figure S8c
γPNA1/2_both_4-nt mismatch top-central	AATTGCCCACCAAGCTGACCACCCAAGGGAGGTTAAGTATGCGGAGAGCTC	Figure S8c
γPNA1/2_both_4-nt mismatch bottom-central	GATCGAGCTCTCCGCATACTTAACCTCCCTTGGGTGGTCAGCTTGGTGGGC	Figure S8c
γPNA1/2_both_5-nt mismatch 5' end top	AATTGCCCACCCTCGTGACGTGGGATCCCTGGTTCTCAATGCGGAGAGCTC	Figure S8c
γPNA1/2_both_5-nt mismatch 5' end bottom	GATCGAGCTCTCCGCATTGAGAACCAGGGATCCCACGTCACGAGGGTGGGC	Figure S8c
γPNA1/2_both_7-nt mismatch 5' end top	AATTGCCCACCCTCGTGTGGTGGGATCCCTCCTTCTCAATGCGGAGAGCTC	Figure S8c
γPNA1/2_both_7-nt mismatch 5' end bottom	GATCGAGCTCTCCGCATTGAGAAGGAGGGATCCCACCACACGAGGGTGGGC	Figure S8c
γPNA1/2_both_9-nt mismatch 5' end top	AATTGCCCACCCTCGACTGGTGGGATCCCTCCAACTCAATGCGGAGAGCTC	Figure S8c
γPNA1/2_both_9-nt mismatch 5' end bottom	GATCGAGCTCTCCGCATTGAGTTGGAGGGATCCCACCAGTCGAGGGTGGGC	Figure S8c
γPNA1/2_both_11-nt mismatch 5' end top	AATTGCCCACCCTGCACTGGTGGGATCCCTCCAAGACAATGCGGAGAGCTC	Figure S8c
γPNA1/2_both_11-nt mismatch 5' end bottom	GATCGAGCTCTCCGCATTGTCTTGGAGGGATCCCACCAGTGCAGGGTGGGC	Figure S8c
γPNA1/2_both_5-nt mismatch 3' end top	AATTGGGGTGCCTCGTGACCACCCAAGGGAGGTTCTCAATCGCCTGAGCTC	Figure S8c
γPNA1/2_both_5-nt mismatch 3' end bottom	GATCGAGCTCAGGCGATTGAGAACCTCCCTTGGGTGGTCACGAGGCACCCC	Figure S8c
γPNA1/2_both_7-nt mismatch 3' end top	AATTGGGGTGGGTCGTGACCACCCAAGGGAGGTTCTCATACGCCTGAGCTC	Figure S8c
γPNA1/2_both_7-nt mismatch 3' end bottom	GATCGAGCTCAGGCGTATGAGAACCTCCCTTGGGTGGTCACGACCCACCCC	Figure S8c

γPNA1/2_both_9-nt mismatch 3' end top	AATTGGGGTGGGAGGTGACCACCCAAGGGAGGTTCTGTTACGCCTGAGCTC	Figure S8c
γPNA1/2_both_9-nt mismatch 3' end bottom	GATCGAGCTCAGGCGTAACAGAACCTCCCTTGGGTGGTCACCTCCCACCCC	Figure S8c
γPNA1/2_both_11-nt mismatch 3' end top	AATTGGGGTGGGAGCAGACCACCCAAGGGAGGTTGAGTTACGCCTGAGCTC	Figure S8c
γPNA1/2_both_11-nt mismatch 3' end bottom	GATCGAGCTCAGGCGTAACTCAACCTCCCTTGGGTGGTCTGCTCCCACCCC	Figure S8c
γPNA1/2_non-consecutive mismatch_2-nt top	AATTGCCCACCGTGGTGACCACCCAAGGGAGGTACACAATGCGGAGAGCTC	Figure S8c
γPNA1/2_non-consecutive mismatch_2-nt bottom	GATCGAGCTCTCCGCATTGTGTACCTCCCTTGGGTGGTCACCACGGTGGGC	Figure S8c
γPNA1/2_non-consecutive mismatch_4-nt top	AATTGCCCAGCGTGGAGACCACCCAAGGGAGCTACACTATGCGGAGAGCTC	Figure S8c
γPNA1/2_non-consecutive mismatch_4-nt bottom	GATCGAGCTCTCCGCATAGTGTAGCTCCCTTGGGTGGTCTCCACGCTGGGC	Figure S8c
γPNA1/2_non-consecutive mismatch_6-nt top	AATTGCCGAGCGTGGAGTCCACCCAAGGGTGCTACACTAAGCGGAGAGCTC	Figure S8c
γPNA1/2_non-consecutive mismatch_6-nt bottom	GATCGAGCTCTCCGCTTAGTGTAGCACCCTTGGGTGGACTCCACGCTCGGC	Figure S8c
γPNA1/2_non-consecutive mismatch_8-nt top	AATTGGCGAGCGTGGAGTCGACCCAAGCGTGCTACACTAAGGGGAGAGCTC	Figure S8c
γPNA1/2_non-consecutive mismatch_8-nt bottom	GATCGAGCTCTCCCCTTAGTGTAGCACGCTTGGGTCGACTCCACGCTCGCC	Figure S8c
γPNA1/2_0-nt mismatch target_top	AATTGCCCACCCTCGTGACCACCCAAGGGAGGTTCTCAATGCGGAGAGCTC	Figure S8c
γPNA1/2_0-nt mismatch target_top	GATCGAGCTCTCCGCATTGAGAACCTCCCTTGGGTGGTCACGAGGGTGGGC	Figure S8c
